# Boosting Interfacial Polarization Through Heterointerface Engineering in MXene/Graphene Intercalated-Based Microspheres for Electromagnetic Wave Absorption

**DOI:** 10.1007/s40820-023-01123-4

**Published:** 2023-06-07

**Authors:** Ge Wang, Changfeng Li, Diana Estevez, Peng Xu, Mengyue Peng, Huijie Wei, Faxiang Qin

**Affiliations:** 1 Institute for Composites Science Innovation (InCSI), School of Materials Science and Engineering, Zhejiang University, 38 Zheda Road, Hangzhou, 310027 People’s Republic of China; 2 Ningbo Institute of Technology, Zhejiang University, 1 Qianhu South Rd, Ningbo, 315100 People’s Republic of China; 3 Foshan (Southern China) Institute for New Materials, Foshan, People’s Republic of China

**Keywords:** MXene, Hierarchical microspheres, Interfacial polarization, Spray-freeze-drying, Microwave absorption

## Abstract

Multi-layer 2D material assemblies provide a great number of interfaces beneficial for electromagnetic wave absorption. However, avoiding agglomeration and achieving layer-by-layer ordered intercalation remain challenging. Here, 3D reduced graphene oxide (rGO)/MXene/TiO_2_/Fe_2_C lightweight porous microspheres with periodical intercalated structures and pronounced interfacial effects were constructed by spray-freeze-drying and microwave irradiation based on the Maxwell–Wagner effect. Such approach reinforced interfacial effects via defects introduction, porous skeleton, multi-layer assembly and multi-component system, leading to synergistic loss mechanisms. The abundant 2D/2D/0D/0D intercalated heterojunctions in the microspheres provide a high density of polarization charges while generating abundant polarization sites, resulting in boosted interfacial polarization, which is verified by CST Microwave Studio simulations. By precisely tuning the 2D nanosheets intercalation in the heterostructures, both the polarization loss and impedance matching improve significantly. At a low filler loading of 5 wt%, the polarization loss rate exceeds 70%, and a minimum reflection loss (*RL*_min_) of −67.4 dB can be achieved. Moreover, radar cross-section simulations further confirm the attenuation ability of the optimized porous microspheres. These results not only provide novel insights into understanding and enhancing interfacial effects, but also constitute an attractive platform for implementing heterointerface engineering based on customized 2D hierarchical architectures.

**Supplementary Information:**

The online version contains supplementary material available at 10.1007/s40820-023-01123-4.

## Highlights

- rGO/MXene/TiO_2_/Fe_2_C heterointerface porous microspheres prepared via scalable method to boost polarization.
- Customization of hierarchical structure by precisely tuning 2D rGO/MXene intercalation.
- Optimal reflection loss of -67.4 dB and EAB = 5.47 GHz at low filler loading of 5 wt%. Simulations showed the benefits of 2D nanosheets intercalation on polarization loss.

**Supplementary Information:**

The online version contains supplementary material available at 10.1007/s40820-023-01123-4.

## Graphical abstract

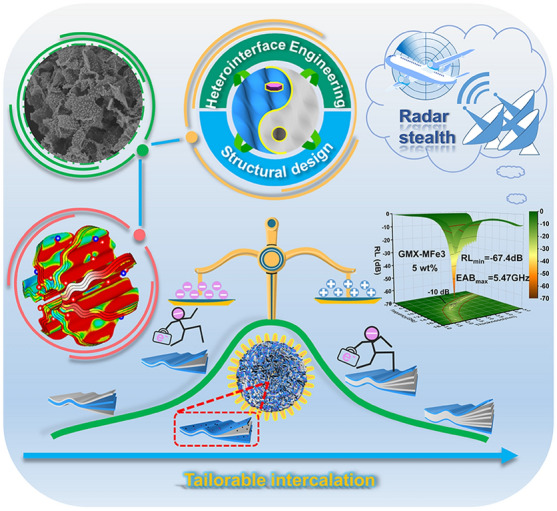

## Introduction

With the development of information technology and radar detection, electromagnetic wave absorbing (EMA) materials play an increasingly significant role in 5G communications, human health, and radar cross section (RCS) reduction [1]. Typically, an ideal EMA material should comply with the requirements of thin thickness, light weight, broadband absorption, and high loss [2–4]. However, since it is difficult for a single component to meet such requirements simultaneously, multi-component structural design across different scales is particularly important [5, 6]. Therefore, rationally designed heterostructures have currently risen as high-efficiency microwave absorbers [7–9].

Of particular interest is the heterointerface formed between two dissimilar materials, in which the asymmetry of electronic and crystal structures affords a spectrum of remarkable characteristics. The characteristics of the heterointerface are governed by the Maxwell–Wagner effect (MWE), which can be applied from the macro- to nanoscale level [10, 11]. With respect to electromagnetic properties, the heterointerface can influence the dielectric and magnetic behaviors through responsive modes such as energy band alignment [12], space charge distribution [11], electron transport [13], lattice defects [14], lattice strain [15], and pegging effects [16]. Both impedance matching and energy dissipation are modulated via those modes. As such, heterointerface engineering has recently emerged as a remarkable strategy for designing high-efficiency and stimuli-responsive EMA materials. Major optimization strategies for heterointerface engineering include composition regulation and structural design [17, 18]. In particular, a rational multi-component heterostructure design can maximize both the interface area and interfacial effects when the role of composition control is limited [8, 10]. 2D materials, especially graphene and MXene, show unique advantages in the field of EMA due to their ultrathin thickness, unique energy band structure and electronic properties, flexible assembly, and tunable dielectric properties [19–22]. MXene is a series of novel 2D transition metal carbides/nitrides/carbonitrides, typically fabricated by selectively etching the metal layer in the MAX phase precursor [23]. Graphene has an ultrathin honeycomb crystal structure consisting of sp^2^ hybridized carbon monolayers [24]. The abundance of reactive surface groups makes graphene and MXene units susceptible to link/hybridize with other materials, which makes them show great potential for the design of heterointerfaces [17]. Besides, compared with 0D nanoparticles and 1D nanowires, 2D nanosheets facilitate the construction of broad-contact interfaces. Especially, 2D/2D contact can fully exploit interfacial effects by maximizing the loading and contact areas [17, 25]. Designing multi-scale heterostructures incorporating dielectric/magnetic components can also trigger stronger polarization loss and optimize EMA performance [1, 26, 27]. However, the assembly of 2D materials such as graphene and MXene is restrained by self-stacking of lamellae and consequently, there are still challenges in understanding and reinforcing interfacial effects from both micro and nano perspectives.

From the view of structural design, 3D porous structures constructed by freeze-drying can enhance interfacial effects while reducing lamellae stacking [5, 28–30]. For instance, the sacrificial template method combined with freeze-drying and heat treatment have been used to encapsulate rGO on the surface of hollow MXene spheres to form a 3D porous structure [31]. The material exhibited efficient EMA performance since such structures produce abundant heterointerfaces facilitating multiple scattering. Compared with the sacrificial template method which is complex and prone to residues after template removal, spray-freeze-drying is promising for assembling 2D materials into multi-dimensional hierarchical structures. 3D MXene hollow spheres with non-stacked structures were designed by spray-lyophilization method [32]. By dispersing the solution into small droplets and freezing them rapidly, 2D MXene nanosheets can be assembled into 3D structures under the action of hydrogen bond pulling and ice crystal extrusion. Microwave-assisted synthesis has also been implemented in the construction of hierarchical structures [33–35] as an efficient strategy to accelerate the reaction process and achieve homogenous heating. Therefore, controlled assembly of multiple low-dimensional structural units into 3D porous structures can be explored based on the combination of these two preparation methods to maximize contact areas and interfacial effects.

In recent years, much attention has been paid to promoting interfacial polarization through the rational design of heterostructures. For example, Wu et al. [7] designed Ti_3_C_2_T_x_/MoS_2_ self-rolling rod-based foams to promote interfacial polarization loss by increasing the heterogeneous surface area, ultimately achieving enhanced EMA performance. Another approach included a PNM (porous skeleton, nanostructure, multilayer construction) design strategy to boost the interfacial polarization of squid pen derived *β*-chitin/carbon nano-onions/Ni–P hierarchical aerogel [10]. Che et al. [25] achieved continuous heterointerfaces and enhanced magnetic coupling by introducing rGO/CoNi nanosheets into MXene films improving the electromagnetic wave (EMW) loss. An interfacial polarization dominated loss mechanism in hierarchical core–shell SiCnws@SiO_2_-carbon foam hybrid composites has also been proposed [36]. However, these works mainly focused on enhancing polarization loss via enlarging the effective heterointerface area (EHA) rather than the heterointerface charge density (HCD) which also makes an important contribution [37]. In fact, precise tailoring of heterostructures and its effect on the interface charge accumulation have rarely been reported.

Here, 3D porous rGO/MXene/TiO_2_/Fe_2_C hierarchical microspheres were successfully fabricated based on a heterointerface engineering approach guided by the MWE. In terms of structural design at the microscopic level, the controllable self-assembly of 2D GO and MXene was achieved by a scalable spray-freeze-drying method, resulting in porous microspheres composed of 2D/2D GO/MXene intercalated structural units. The subsequent microwave irradiation process constructed the 2D/2D/0D/0D intercalated heterostructure by introducing semiconducting TiO_2_ and magnetic Fe_2_C nanoparticles into the structural units of the microspheres. The introduction of the nanoparticles increased the interfacial polarization charge density while enlarging the heterogeneous surface area. Subsequently, MWE was used to elucidate the charge accumulation behavior affected by different intercalation structures, which resulted in regulable polarization characteristics. The enhancement of interfacial polarization triggered by such effect was verified by simulations in CST microwave studio. By adjusting the ratio of GO and MXene in the precursors, the intercalation of the 2D materials in the structural units was precisely designed modulating the heterointerface to meet the EMW attenuation requirements. The method proposed in this work can provide insight into the precise construction of multi-layer structures, opening new avenues for enhancement and optimization of interfacial effects in electromagnetic absorbers.

## Theory and Experimental

### MXene/rGO Heterointerface (MGH) Model

The polarization intensity at the heterointerface ($P_{interface}$) is affected by the area of the polarization interface ($S$) and the polarization charge density of the heterointerface ($\sigma_{f}$) [7]:

1 $$ P_{interface} \propto S \cdot \sigma_{f} $$

According to MWE, the accumulated interfacial polarized charge density ($\sigma_{f}$) is related not only to the dielectric properties (*ε, σ*) of the two phases at the interface, but also to the thickness of the two phases (Section S1):

2 $$ \sigma_{f} = \smallint \left[ {J_{1} - J_{2} } \right]dt = \frac{{\varepsilon_{2} \sigma_{1} - \varepsilon_{1} \sigma_{2} }}{{\sigma_{1} d_{2} + \sigma_{2} d_{1} }} \cdot U $$

3 $$ = \frac{{\varepsilon_{2} \sigma_{1} - \varepsilon_{1} \sigma_{2} }}{{d_{1} \left( {\sigma_{1} \gamma + \sigma_{2} } \right)}} \cdot U $$

where $d_{1}$*,* $\varepsilon_{1}$*,* $\sigma_{1}$*,*$d_{2}$*,* $\varepsilon_{2}$*,* $\sigma_{2}$ are the thickness and dielectric parameters of materials forming the heterointerface respectively, $\gamma = \frac{{d_{2} }}{{d_{1} }}$ is the defined thickness factor.

Focusing on the improvement of interfacial polarization intensity, a face-to-face heterointerface model was constructed choosing MXene and rGO as template materials. Such study will guide the experimental design and maximize the triggering of interfacial polarization. Based on MWE, the maximum interfacial area and the highest polarized charge density were obtained when the MXene and rGO phases form a structure with periodic alternating 2D/2D intercalation (minimum $d_{1}$, $d_{2}$). Moreover, the thickness factor indicates that the periodic intercalation mode of MXene/rGO nanosheets also affects the mechanism of charge accumulation and migration at the heterointerface [38]. In the following sections, the above analysis will be demonstrated based on experiments and simulations.

### Experimental Section

#### Materials

Expanded graphite (EG, 80 mesh, ∼50 μm) was purchased from Qingdao Santong Graphite Co., Ltd. China. Ti_3_AlC_2_ powders (> 99.5 wt% purity) were purchased from Foshan Xin Xi Technology Co., Ltd. China. Carbon fiber was purchased from Toray company. Lithium fluoride (LiF) and ferrocene were purchased from Aladdin Industrial Corporation. Potassium permanganate (KMnO_4_), hydrogen peroxide (H_2_O_2_), sulfuric acid, hydrochloric acid (HCl) and dichloromethane were obtained from Sinopharm Chemical Reagent Co., Ltd. China. All chemical reagents were of analytical grade and not further purified.

#### Preparation of MXene and GO Suspensions

The Al layers can be removed by selective wet chemical etching of Ti_3_AlC_2_ (Section S2). Firstly, 2 g Ti_3_AlC_2_ was gradually added to a pre-stirred mixture of LiF (3 g) and HCl (9 M 40 mL) within 10 min, and stirred at 40 °C for 36 h. Secondly, the mixture was repeatedly washed and centrifuged with deionized water at a speed of 3,500 rpm for 2 min until pH value became ∼7. Thirdly, the precipitate was redispersed in deionized water in an ice bath and sonicated for 1 h under Ar atmosphere. Then, the suspension was centrifuged at 3,500 rpm for one hour to discard the precipitate. Finally, a black concentrated supernatant of delaminated Ti_3_C_2_T_x_ nanosheets was successfully obtained. The graphene oxide (GO) suspension was prepared by the modified Hummers’ method [39]. The concentrations of GO and MXene suspensions were obtained by measuring the mass of the part left after freeze-drying divided by the volume of the suspension.

#### Fabrication of GO/ MXene Microspheres

Porous GO/MXene microspheres were synthesized by low-temperature spray-freeze-drying procedure. GO suspension (18.75 mL, 8 mg mL^−1^) and MXene suspension (31.25 mL, 4.8 mg mL^−1^) were added to deionized water (50 mL) and stirred at a speed of 500 rpm for 30 min. The obtained dispersion was nebulized through an atomization nozzle using a commercial electric sprayer and frozen immediately when sprayed into liquid nitrogen. Finally, the frozen droplets were dried in a vacuum freezing dryer for 72 h under 0.1 Pa pressure. With a fixed total mass of MXene and GO (3 mg mL^−1^), GO/MXene microspheres with mass ratios of 3:1, 2:1, 1:1, and 1:2 were obtained, which were labeled as GMX1, GMX2, GMX3, and GMX4, respectively. By controlling the concentration of nanosheets in the precursor solution to 3 mg ml^−1^, pure GO and MXene microspheres were also fabricated by the same procedure and labeled as GOS and MXS, respectively.

#### Microwave Irradiation of GO/MXene Microspheres

To fabricate rGO/MXene/TiO_2_/Fe_2_C microspheres, three bundles of carbon fibers CFs (2 cm in length, 4.5 mg in total) were used as initiator to trigger the arc discharge for microwave irradiation. The aforementioned GO/MXene microspheres (GMX1…etc.) (50 mg), and ferrocene (50 mg) were mixed together in a quartz vial by homogenizers (Shenzhen Zhongyi Technology Co. Ltd.). Then the mixtures were irradiated with microwaves (900 W) for 60 s under Ar atmosphere. After that, the bundle of CFs was removed with tweezers. Finally, the obtained products were washed by dichloromethane to remove the unreacted ferrocene, and then dried in vacuum. The microwave-treated GMX1, GMX2, GMX3 and GMX4 were labeled as GMX-MFe1, GMX-MFe2, GMX-MFe3, GMX-MFe4, respectively. rGO/MXene/TiO_2_ microspheres were also prepared in a similar process without adding ferrocene, which were denoted as GMX-M1, GMX-M2, GMX-M3, GMX-M4, respectively. In turn, microwave irradiated GO microspheres and MXene microspheres were labeled as GOS-M and MXS-M, respectively. Table 1 summarizes the description of the studied samples and their corresponding labeling.

**Table 1:** Prepared microspheres with different GO/MXene ratios and corresponding labeling

Microsphere	GO/MXene ratio			
	3:1	2:1	1:1	1:2
GO/MXene spray-freeze-dried (SFD)	GMX1	GMX2	GMX3	GMX4
GO/MXene (SFD)&Microwave-irradiated (MW)	GMX-M1	GMX-M2	GMX-M3	GMX-M4
GO/MXene (SFD)(MW)&Ferrocene	GMX-MFe1	GMX-MFe2	GMX-MFe3	GMX-MFe4

#### Radar Cross Section (RCS) Simulation

To evaluate the electromagnetic scattering capability under far-field conditions, CST Studio Suite was used to simulate the RCS values of the samples. The constructed model consists of an absorber/paraffin layer with a thickness of 3.1 mm (5 wt% filling ratio) and a perfect electric conductor (PEC) backing with a thickness of 4 mm. In the meantime, the length and width of each layer were set to 300 and 200 mm, respectively. The model was placed on the XOY plane, the linearly polarized EMW was incident from the Z-axis, and the electric polarization was along the X-axis. The RCS value is calculated by the following equation:

4 $$ \sigma \left( {dBm^{2} } \right) = 10\log \left[ {\frac{4\pi S}{{\lambda^{2} }}\left| {\frac{{E_{s} }}{{E_{i} }}} \right|^{2} } \right] $$

where *S* and* λ* denote the area of the simulation model and the EMW wavelength, respectively. And *E*_*s*_ and *E*_*i*_ are the electric field strengths of the scattered and incident EMW, respectively.

#### Characterization

The phases identification of the samples was performed by X-ray diffraction (XRD, Rigaku Smart Lab diffractometer, Japan, with Cu-Kα radiation (1.54178 Å). The chemical structures and bonding states of carbon were investigated by Raman spectroscopy (DXR Smart Raman spectrometer (irradiation wavelength: 532 nm). The chemical element analyses were conducted by X-ray Photoelectron Spectroscopy (XPS, Thermo Scientific K-Alpha). Characteristic vibrational groups were analyzed by Fourier Transform Infrared Spectroscopy (FTIR, Nexus Nicolet 5700 spectrophotometer with KBr pellets). The morphology and structure of the materials were examined by Field Emission Scanning Electron Microscopy (SEM, SU8010, Hitachi) and Transmission Electron Microscopy (TEM, FEI Talos F200s).

The magnetic performance was measured by Vibrating Sample Magnetometer (VSM, Quantum Design, Kratos Analytical Ltd) at room temperature. The dimension of as-prepared GO and MXene nanosheets was measured by a scanning probe microscope (MultiMode, Veeco Instruments Inc, America). The electromagnetic parameters in a frequency range of 2–18 GHz were measured by a vector network analyzer (R & S ZNB20) with coaxial line method. The microspheres (mass fraction of 5 wt%) were uniformly mixed with paraffin and pressed into a standard toroidal shape with an outer diameter of 7 mm and an inner diameter of 3.04 mm.

## Results and Discussion

### Structural and Morphological Properties

**Fig. 1:**
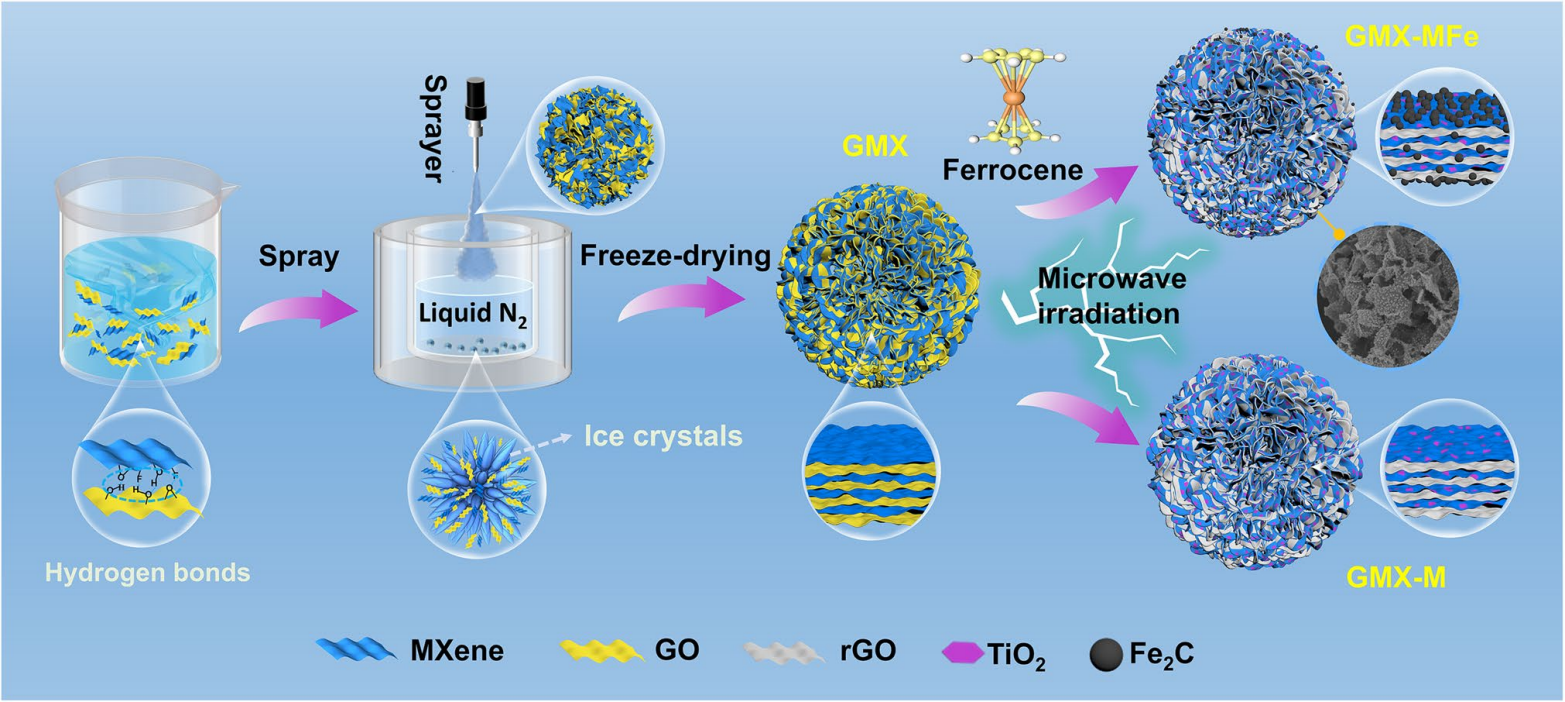
Schematic illustration for the synthesis of spray-freeze-dried GO-MXene GMX, microwave irradiated GO-MXene GMX-M and ferrocene-added GMX-MFe microspheres

Figure 1 illustrates the preparation process and formation mechanism of 3D hierarchical microspheres. Initially, MXene and GO monolayer nanosheets exhibit ultrathin laminar structures (Fig. S3). Due to the presence of a large number of hydrophilic functional groups (= O, –OH, –F), both GO and Ti_3_C_2_T_x_ nanosheets form light-brown and dark-green stable homogeneous dispersions in deionized water, respectively (Fig. S4a-b). The GO and MXene dispersions were then well mixed (Fig. S4c) by stirring due to the presence of hydrogen bonding and Van der Waals forces [29]. Secondly, when the ejected droplets are rapidly frozen by liquid nitrogen, the growing ice fronts tend to expel or entrap the GO and Ti_3_C_2_T_x_ nanosheets. Under such circumstances, the hybrid assemblies formed from both hydrogen bonding and Van der Waals interactions are preserved, establishing 2D/2D intercalated hetero-structural units. Then, these units can assemble between the numerous ice fronts and eventually along the ice crystal boundaries to form 3D porous microspheres [19, 22, 40]. Thirdly, the microspheres were homogenously mixed with CFs and subjected to microwave irradiation under argon atmosphere. Upon irradiation, CFs are used as an effective initiator to trigger the arc discharge [41], which contributes to the rapid heating of GO and MXene and the transfer of microwave energy to ferrocene. The ferrocene then starts to decompose and form metallic iron nuclei. In the meantime, the Fe atoms aggregate and attach to the graphene and MXene substrates, eventually transforming to Fe_2_C [34, 42]. With the addition of MXene, GO undergoes an avalanche of deoxygenation reactions under microwave irradiation [43–45], leading to rGO formation. Finally, the as-prepared microspheres can be easily attracted by magnets. Here, the heterojunction of constituent units for the 3D microspheres can be easily modified by adjusting the mass ratio of GO and MXene, thus enabling the enhancement of the EMA properties through tuning the heterointerfaces.

**Fig. 2:**
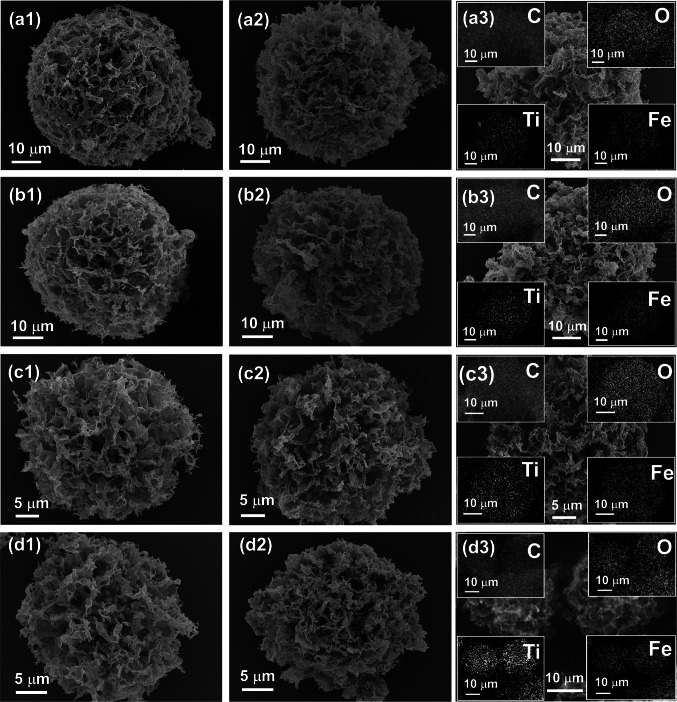
Morphology of microspheres: (**a1**) GMX1, (**a2**) GMX-M1, (**a3**) GMX-MFe1, (**b1**) GMX2, (**b2**) GMX-M2, (**b3**) GMX-MFe2, (**c1**) GMX3, (**c2**) GMX-M3, (**c3**) GMX-MFe3, (**d1**) GMX4, (**d2**) GMX-M4, and (**d3**) GMX-MFe4. The insets are the corresponding element mapping images

From the SEM images in Fig. 2, the microspheres have a porous flower-like morphology. The basic framework of these microspheres is assembled from ‘petal-like’ structural units formed by nanosheets of both 2D materials, leading to rich heterointerfaces and multiple scattering of EMW [46]. Taking GMX3, GMX-M3, and GMX-MFe3 as examples, the particle size of the microspheres is uniform (the average diameter is about 40 μm), and nearly the same before and after microwave irradiation. 3D interconnected structures with porous networks can be observed in all samples. As the content of GO in the precursor solution decreases, the microspheres obtained with a fixed spray power gradually become smaller, which can be attributed to the gelation ability of GO. This is also evidenced by the size of the pure GO and MXene microspheres (Fig. S5). Moreover, the energy dispersive spectroscopy (EDS) mapping scans for magnetic GMX-MFe samples show a uniform distribution of Ti, C, O and Fe elements (insets of Fig. 2a3-d3), indicating that GO and MXene are well mixed and Fe_2_C nanoparticles are also uniformly anchored to the lamellar structural units without severe agglomeration (Fig. S6). The construction of multi-scale structure, porous skeleton and multi-layer structure would strengthen interfacial effects [10].

**Fig. 3:**
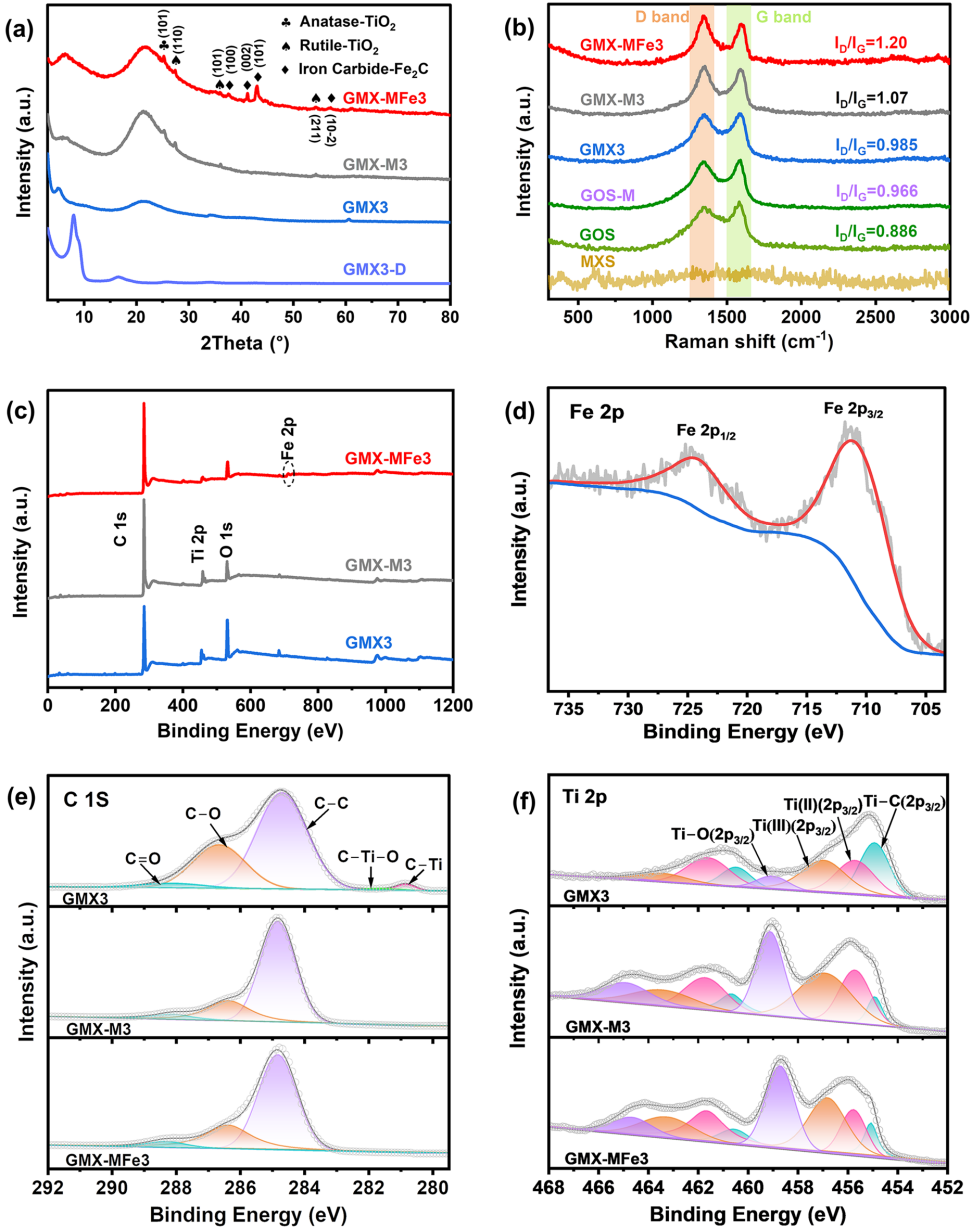
**a** XRD patterns of spray-freeze-dried GMX3, dispersed GMX3-D, microwave-irradiated GMX-M3 and ferrocene-added GMX-MFe3 samples. **b** Raman spectra of MXene microspheres MXS, GO microspheres GOS, microwave-irradiated GOS-M, GMX3, GMX-M3, GMX-MFe3. **c** XPS survey spectrum of GMX3, GMX-M3 and GMX-MFe3. High-resolution XPS spectra **d** Fe 2*p* spectrum of GMX-MFe3. **e** C 1*s* and **f** Ti 2*p* spectra of GMX3, GMX-M3 and GMX-MFe3

Figure 3a shows the XRD patterns of GMX3, GMX3-D, GMX-M3 and GMX-MFe3. To verify the role of spray-freeze-drying process in GO/MXene intercalation, GMX3 dispersed in deionized water and post-heated at 60 °C under vacuum (labelled as GMX3-D) was also analyzed. GOS-D and MXS-D were obtained from GOS and MXS by the same treatments. The GO/MXene microsphere structure can be easily dispersed in water and remains stable even after being disrupted by stirring (Fig. S4d), demonstrating the reversibility of the spray-freeze-drying method for assembling 3D microspheres.

A diffraction peak at 4.98° corresponding to the (002) crystalline plane of MXene can be observed in GMX3 spectrum (Fig. 3a) while the characteristic diffraction peaks of GO (~ 10°) and rGO (~ 26°) [47–49] are not detected. Instead, a broad diffraction peak appears at 21°, which contrasts with the sharp diffraction peak at 9.46° in GO aerogel spectrum (Fig. S7a) ascribed to the (001) plane of GO. This phenomenon could be due to the fact that the layer spacing of GO is more sensitive to solvent/lamella interaction than that of MXene (Fig. S7b). Under such circumstances, the GO nanosheets were unable to be long-range ordered immediately after being frozen by liquid nitrogen, but instead were diffusely distributed, leading to the appearance of a broad diffraction peak. After vacuum drying the dispersion suspension of GOS microspheres, the restacking of GO led to the appearance of the (001) diffraction peak of GO in the XRD spectrum of GOS-D (Fig. S7a). Similarly, after the same treatment of GMX3 microspheres, the restacking of GO and MXene resulted in the appearance of a superposition peak consisting of the MXene (002) crystalline plane and the GO (001) crystalline plane in the XRD spectrum of GMX3-D (Fig. S8) [19].

The above results clearly demonstrate the significant role of spray-freeze-drying process in preventing the random self-restacking of GO and MXene nanosheets. When the small droplets were rapidly frozen in liquid nitrogen, the homogeneous dispersion of GO and MXene nanosheets was maintained because the time was too short to allow random agglomeration [50]. Meanwhile, hierarchical morphologies were formed between GO and MXene nanosheets through hydrogen bonding and Van der Waals forces, facilitating the formation of 2D/2D alternated intercalation heterostructures.

As shown in Fig. 3a, new peaks assigned to rutile TiO_2_ (JCPDS No. 01–070–7347) and anatase TiO_2_ (JCPDS No. 01–070–6826) [51] emerge in GMX-MFe3 spectrum after microwave irradiation, indicating the partial oxidation of MXene to TiO_2_. In addition, the diffraction peaks at 37.58°, 41.24°, 43.06°, and 57.12° confirm the presence of Fe_2_C (JCPDS No. 00–036–1249) [52] resulting from the decomposition of ferrocene. The XRD patterns of GMX-MFe1, GMX-MFe2, and GMX-MFe4 also show the same characteristic peaks (Fig. S9a). The GO is reduced after microwave irradiation, and a characteristic diffraction peak corresponding to (002) plane of rGO is observed at 25.76° in the rGO aerogel spectrum (Fig. S7a). This peak is not present in the GMX-M3 due to GO dispersion in GMX3. Due to the fast freezing and subsequent freeze-drying, the GO structure in the dispersion is preserved, and thus no broad amorphous characteristic peaks of rGO can be found in GOS-M and GMX-M3 samples (Fig. 7a and S7a). Additionally, the FTIR spectra show a diminution of oxygen-containing functional groups in GMX-M3 and GMX-MFe3 after microwave irradiation (Fig. S10a).

In the Raman spectrum of GO microspheres, distinct D and G peaks are observed, while no distinct peaks exist in the MXene microspheres (Fig. 3b). The D band corresponds to the hybridized vibrational mode of *sp*^3^ defects and the G band represents the vibrations of *sp*^2^ carbon atoms [29]. In general, the intensity ratio of D peak to G peak (*I*_D_/*I*_G_) sheds light on the reduction degree of GO and indicates the number of defects in the carbon material [48, 53]. After microwave irradiation, the *I*_D_/*I*_G_ ratios of GOS-M and GMX-MFe3 increase compared to those of GOS and GMX3, and are more significant when the microspheres contain both MXene and graphene oxide. Such increase implies that the reduction of GO to rGO after microwave irradiation generates a large number of defects in the hierarchical microspheres, which would favor the formation of abundant heterointerfaces. These defects enhance dipole polarization and facilitate optimization of impedance matching, thus promoting the loss of electromagnetic waves [47].

**Fig. 4:**
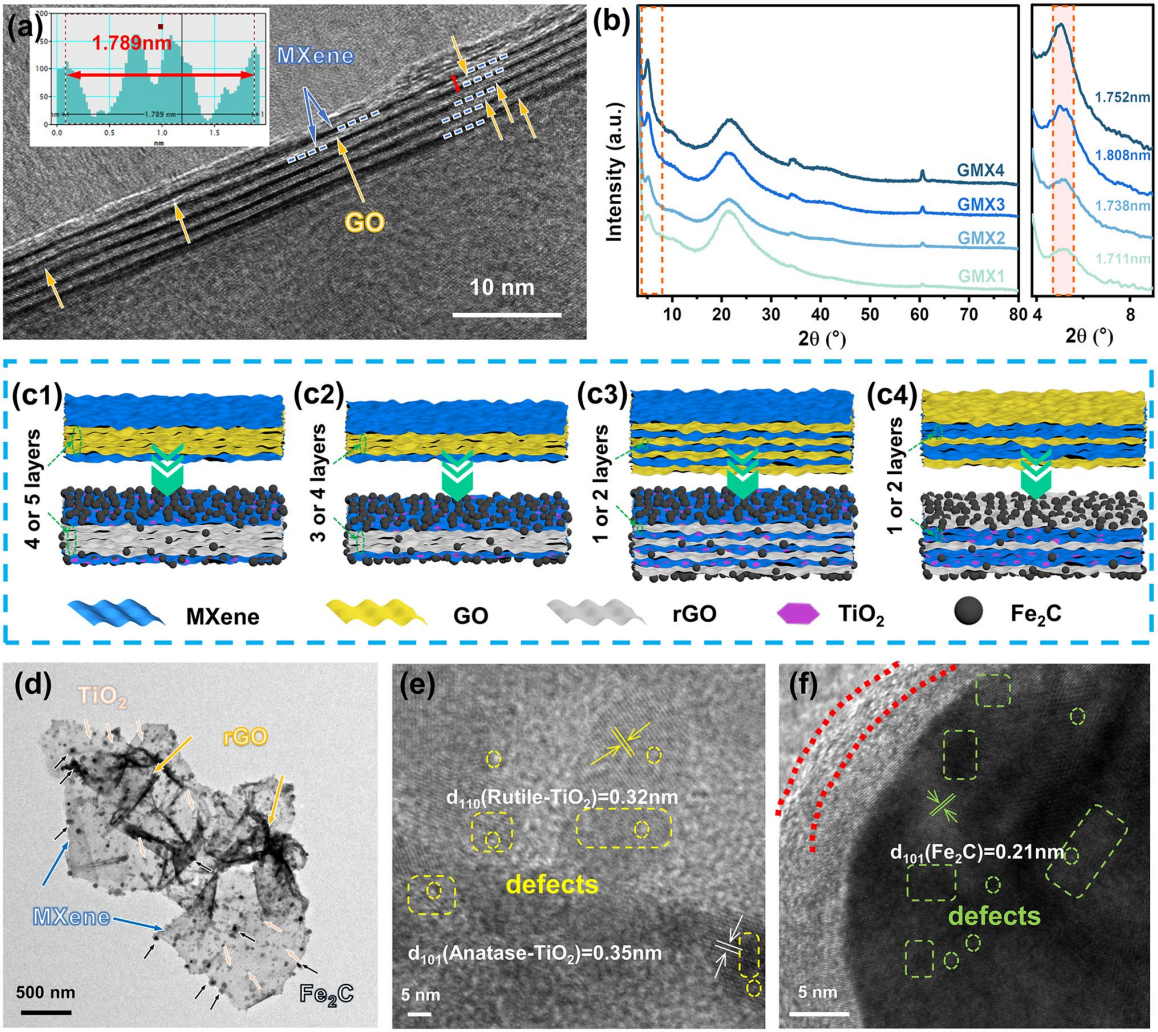
**a** HRTEM image of GMX3, and the layer spacing of MXene along the red line (inset). **b** XRD patterns and layer spacing values of the GMX with different GO to MXene ratios. Schematic illustration of structural units showing the variation with GO/MXene ratio for different microspheres: **c1** GMX1 and GMX-MFe1, **c2** GMX2 and GMX-MFe2, **c3** GMX3 and GMX-MFe3, **c4** GMX4 and GMX-MFe4. **d** TEM image of the structural units for GMX-MFe3. HRTEM images of lattice defects in **e** TiO_2_ and **f** Fe_2_C

The chemical composition of MXene and GO nanosheets and their interaction during microwave irradiation can be further explored by XPS characterization. All XPS spectra show the presence of C, O and Ti (Fig. 4a). As shown in Fig. 3d, GMX-MFe3 exhibits characteristic peaks ascribed to Fe 2*p* (711.3 eV for Fe 2*p*_3/2_, 724.5 eV for Fe 2*p*_1/2_ [54]), indicating the successful anchoring of Fe_2_C on the lamellar structural units of the microspheres. High-resolution XPS spectra of C 1*s* and Ti 2*p* are shown in Fig. 3e–f. The C 1*s* spectra of GMX3 can be deconvoluted into five peaks corresponding to C-Ti (281.8 eV), C-Ti–O (282.4 eV), C–C (284.8 eV), C-O (286.2 eV), and C = O (288.1 eV) bonds [49, 55]. In the Ti 2*p* spectrum, the Ti 2*p*_3/2_ at 454.9, 455.9, 457.0 and 458.9 eV can be assigned to Ti-C, Ti (II), Ti (III) and Ti–O bonds, respectively [55–57]. As seen in Fig. 3e, after microwave irradiation, the intensity of C–O and O = C–O peaks decreases, the C–Ti and Ti–C–O peaks disappear while the intensity of Ti–O peak increases. The XPS results clearly illustrate the deoxygenation of GO and partial oxidation of MXene after microwave irradiation, which is consistent with previous XRD and Raman characterization results [58, 59]. Interestingly, GO deoxygenation occurs after microwave irradiation of GOS (Fig. S10b), while there is no obvious change for Ti–O peaks of MXS (Fig. S10d). This means that some MXene nanosheets undergo the redox process with GO layers when the GO/MXene microspheres are exposed to microwave irradiation. Specifically, MXene in the intercalated structure participates in the reduction of GO via transferring oxygen from GO to MXene [53, 60–62]. The reduction of GO increases the conductive loss while the partial oxidation of MXene leads to decrease in conductivity [24]. As a result, the redox process can minimize the difference in dielectric properties between GO and MXene nanosheets, providing them with complementary matching properties [1].

High resolution TEM (HRTEM) was used to observe the microstructure of the microspheres. Figure 4a shows the alternated intercalation heterostructure of monolayer GO and MXene, where the MXene nanosheets are separated by GO. The 2D/2D surface contact maximizes the contact area and fully reinforces interfacial effects [17]. The spacing between MXene layers is 1.789 nm (inset in Fig. 4a), which is much larger compared to that in MXene microspheres (1.46 nm, Fig. S11).

XRD tests for different microspheres were performed to assess the MXene layer spacing variation with the introduction of GO. The MXene microspheres show a strong (002) diffraction peak at 6.2°, corresponding to the layer spacing of 1.429 nm (Fig. S7b). After intercalation self-assembly of different ratios of GO and MXene nanosheets, the (002) peak of MXene shifts from 6.2 to 5.16° for GMX1, 5.08° for GMX2, 4.98° for GMX3 and 5.04° for GMX4, and consequently the corresponding layer spacings increase to 1.711, 1.738, 1.808, and 1.752 nm, respectively (Fig. 4b). The layer spacing decreases slightly due to excess Ti_3_C_2_T_x_ when the GO/MXene ratio increases from 1:1 to 1:2. The variation in layer spacing reflects the different intercalation periodicities of GO and MXene in the GMX structural units (Fig. 4c1-c4 and Section S6), which would be preserved in the GMX-M and GMX-MFe samples. Such heterointerface modulation will eventually affect the EMA performance [26].

The TEM image of the structural units based on GMX-MFe3 microspheres shows that the lamellar units consist of alternated intercalated layers of MXene and rGO nanosheets (Fig. 4d). There are numerous wrinkled areas in rGO sheets, and TiO_2_ nanoparticles are uniformly distributed in the MXene sheets. In addition, Fe_2_C nanoparticles are homogeneously decorated on the ‘petals’ of the translucent microspheres demonstrating the superiority of microwave irradiation in halting nanoparticle agglomeration. Figure 4e shows the HRTEM lattice image of TiO_2_, in which the lattice distances of 0.35 nm and 0.32 nm match the (101) plane and (110) plane of the anatase and rutile structures, respectively [51]. In Fig. 4f, the lattice distance of 0.21 nm corresponds to the (101) crystal plane of Fe_2_C [63]. Besides, Fe_2_C particles are encapsulated in an amorphous carbon shell, exhibiting a unique core–shell structure. These rGO/MXene/TiO_2_/Fe_2_C (2D/2D/0D/0D) heterojunctions provide sufficient contact area for numerous heterointerfaces, which can significantly increase the interfacial polarization loss [26]. Furthermore, as shown in Fig. 4d–f, there are numerous lattice defects such as lattice distortion (square marked regions) and vacancies (round marked regions) in TiO_2_ and Fe_2_C nanoparticles. These lattice mismatches affect charge transport and trigger charge aggregation, generating spatial dipole moments to enhance polarization relaxation, which contributes to EMW attenuation [17, 64]

### Heterointerface Model

**Fig. 5:**
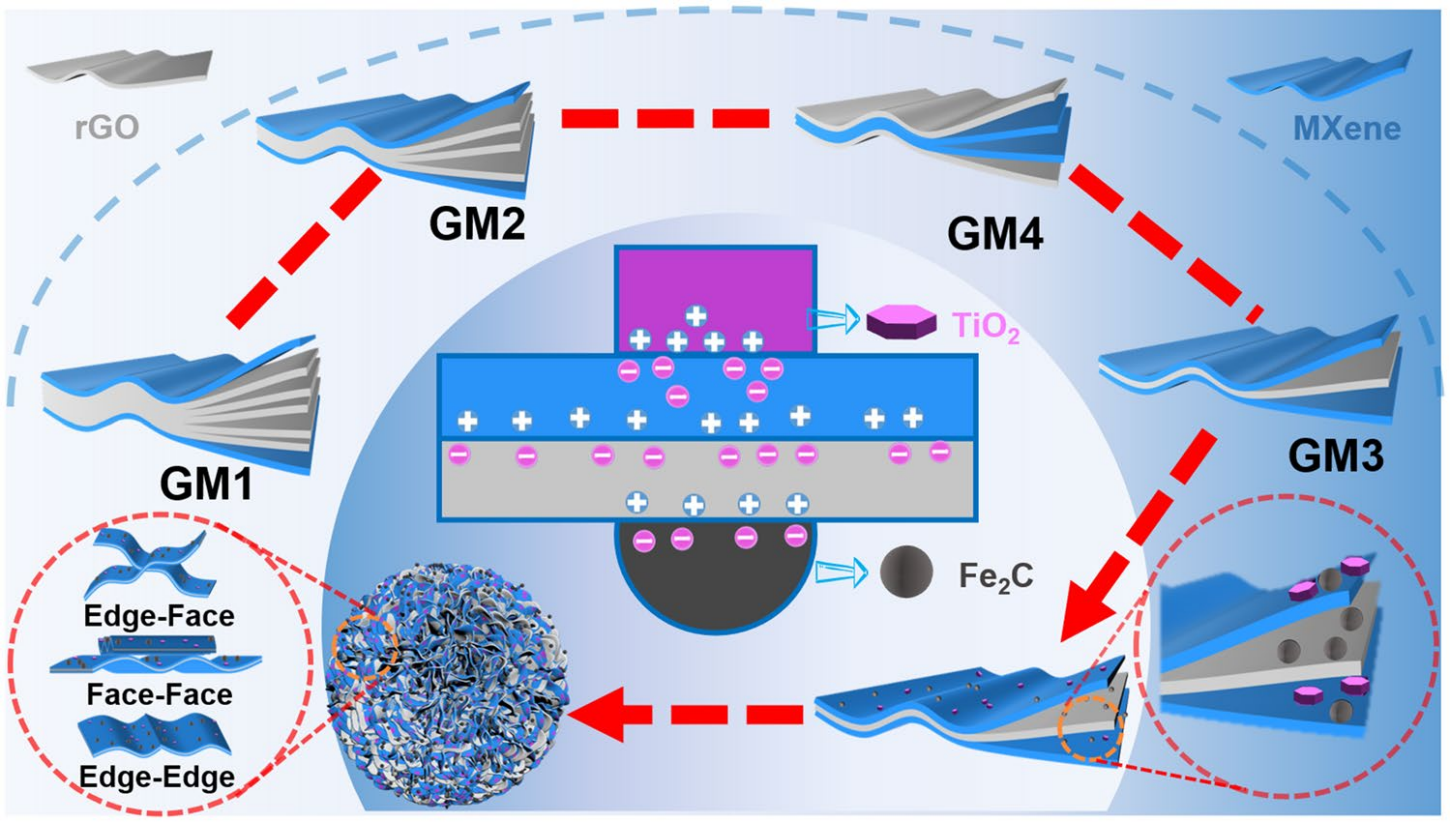
Schematic diagram of MXene/rGO heterointerface (MGH) engineering model

**Fig. 6:**
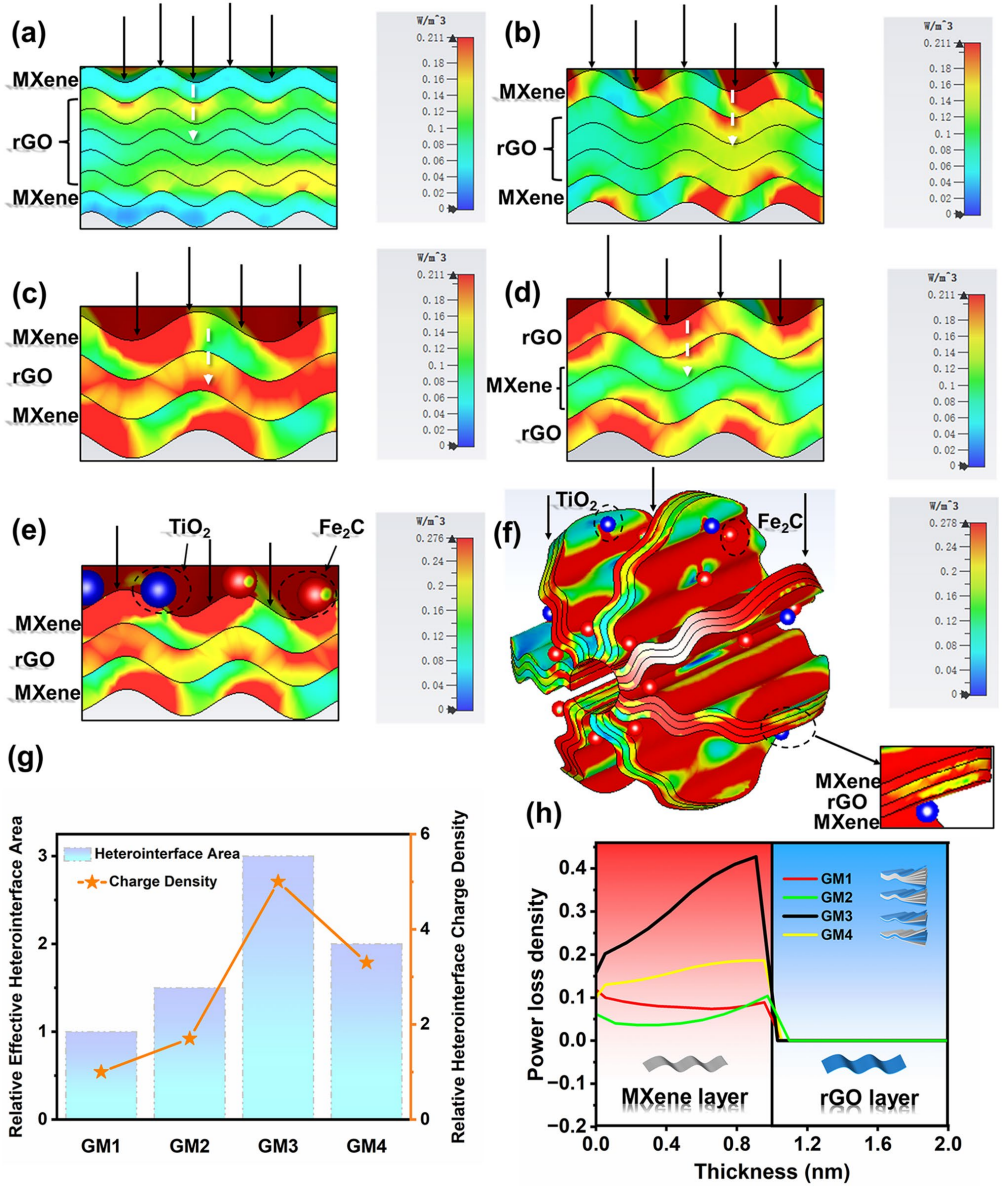
CST simulations of power loss distribution, **a** GM1, **b** GM2, **c** GM3, **d** GM4, **e** GM3-particles, **f** GM3-particles-microspheres. **g** Relative effective heterointerface area (EHA) and charge density (HCD) of GM1-GM4. **h** The value of power loss density from GM1 to GM4 extracted from the white dotted line in Fig. 6a-e

To analyze the interfacial polarization intensity of different intercalated units, we propose four simplified MXene/rGO intercalation models based on the composition design and microstructure characterization, namely GM1 to GM4 (Fig. 5). According to the MGH model,the relative EHA and HCD are calculated at a fixed MXene/rGO nanosheets amount (Fig. 6g and Section S7). From GM1 to GM4, the relative EHA and HCD first increase and then decrease, which closely relate to the intercalation modes. The largest relative EHA and HCD are achieved in GM3, resulting in the highest interfacial polarization intensity.

From the perspective of microscopic charge migration mechanism, the work function difference between MXene and rGO leads to charge migration and aggregation at the heterointerface [17, 62]. The accumulation of positive and negative charges near the heterointerface forms a space charge zone and bends upward the conduction band (CB)/valence band (VB) to form a barrier inhibiting electron reflux and causing loss [65]. Moreover, from Fig. S15, as the number of rGO layers increases (from monolayer to more than 5 layers), the work function difference between rGO and MXene gradually decreases [66, 67]. This means that the heterointerface composed of monolayer MXene and rGO has the highest interfacial polarization loss (thus, the highest interfacial energy barrier). Furthermore, compared to other intercalation models, the 2D/2D ultra-thin interfaces in the GM3 model shorten the transmission distance of charges and reduce the recombination rate in the process of migration. This results in higher polarization charge accumulation at the interface [68].

After the introduction of nanoparticles, more charge aggregation sites can be created by generating more interfaces and defects. At the same time, the work function difference between TiO_2_/Fe_2_C and MXene/rGO contributes to the generation of quasi-metal–semiconductor interfaces (Fig. S15), which forms the Schottky barrier [69, 70]. This facilitates the charge migration and aggregation at the heterointerface, resulting in strong polarization loss. In the microspheres, face-to-face contact, face-to-edge contact and edge-to-edge contact of structural units result in richer heterogeneous surface types and higher interfacial polarization loss. The above conclusion further confirms the interpretations of the MGH model from the microscopic point of view and demonstrates that heterointerface engineering based on intercalated structures effectively enhances the polarization loss.

### CST Simulation of Polarization Intensity

CST Microwave Studio 2020 was implemented to further verify the level of interfacial effects in the intercalated GM1-GM4 structures and after the incorporation of the particles and assembly of the spheres (Section S8). The power loss distribution under the same incident EMW is shown in Fig. 6. The power loss first occurs at the heterointerface between nanosheets, and then expands to the interior of the intercalation (Video S1). From Fig. 6a–d and h, compared with other intercalation models, the ideal intercalation model GM3 has the largest power loss density at the interface, which is highly consistent with our MGH model analysis. After the introduction of TiO_2_ and Fe_2_C particles, the polarization effect increases and the power loss intensity between nanosheets and Fe_2_C is stronger than with TiO_2_ (Fig. 6e). The power loss intensity is further improved after the intercalated structural units are assembled into microspheres (Fig. 6f), which is consistent with the theoretical analysis. The electromagnetic parameters and absorbing properties will be analyzed in the next section to supplement these conclusions.

### Microwave Absorption Properties

**Fig. 7:**
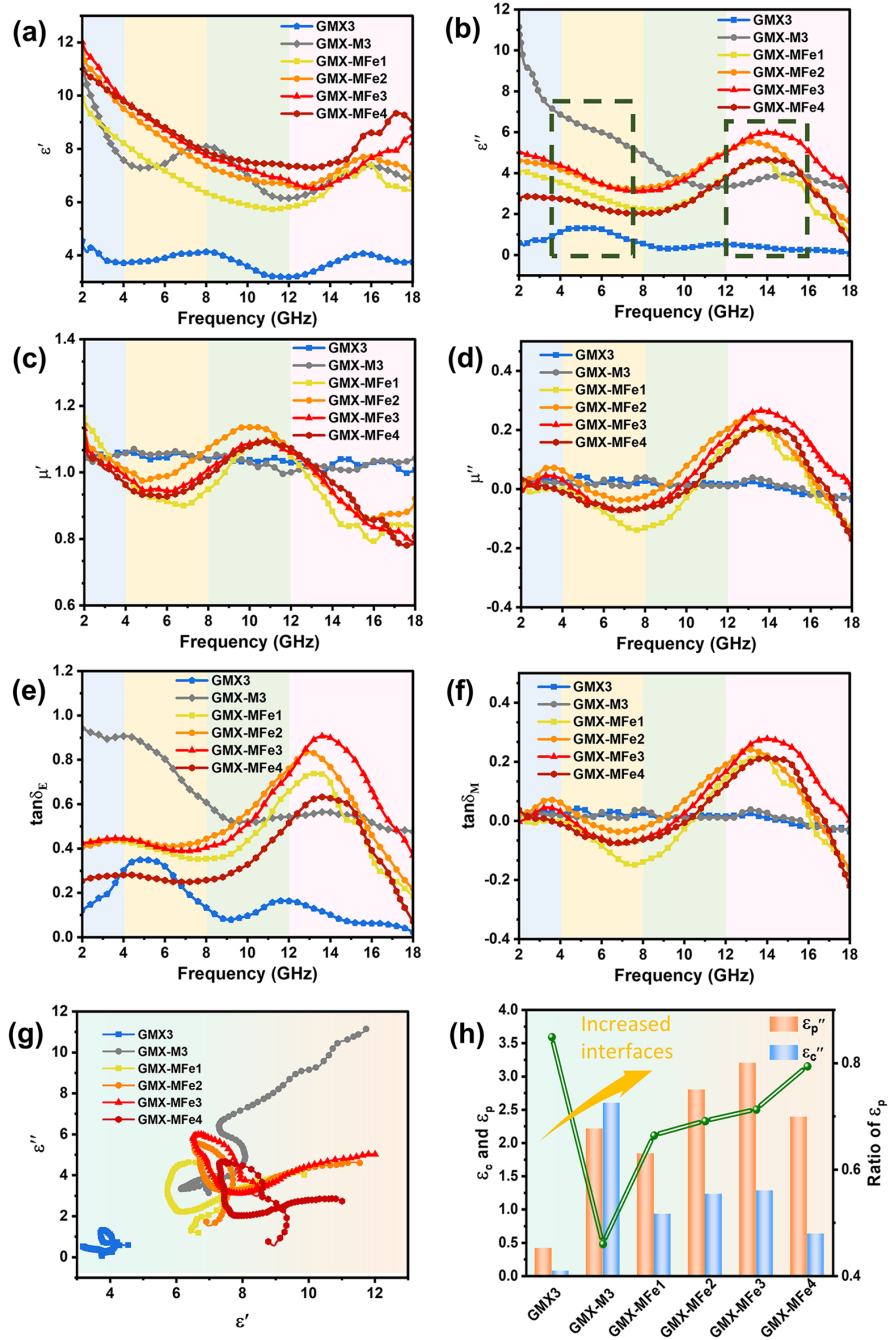
Electromagnetic parameters of GMX3, GMX-M3 and GMX-MFe series, including **a** real (*ε′*) and **b** imaginary part (*ε′′*) of permittivity, **c** real (*μ′*) and **d** imaginary part (*μ′′*) of permeability, **e** dielectric loss tangent tan *δ*_*E*_, and **f** magnetic loss tangent tan *δ*_*M*_. **g** Cole–Cole semicircles of GMX3, GMX-M3 and GMX-MFe series. **h** The conductive and polarization loss for all the samples

To further reveal the mechanism of EMW loss, the electromagnetic parameters of the samples were investigated. The dielectric loss characteristics in 2–18 GHz are caused by multi-polarization composed of interfacial polarization (space charge polarization), dipole polarization, and conductive loss. Electronic and ionic polarizations have no contribution to the complex permittivity in 2–18 GHz frequency band [9, 71]. As shown in Fig. 7a–b, compared with GMX-M3 and GMX-MFe samples, GMX3 exhibits lower *ε'* (electric storage capacity) and *ε''* (electric loss capacity) values, which is attributed to the insulating GO nanosheets blocking the continuous electron transport between MXene nanosheets in the intercalated structures [6]. Meanwhile, without the 0D particles bringing heterojunctions and defects, GMX3 exhibits weak polarization relaxation characteristics (smooth fluctuation in the complex permittivity curves). For GMX-M3, the mean values of complex permittivity increase, and more resonant peaks appear. Compared with GMX3, the reduction of GO nanosheets provides conductive paths for charge migration while the in-situ generation of TiO_2_ particles on MXene nanosheets contributes defects and heterojunctions, which promotes conductive and polarization losses in GMX-M3.

After the introduction of Fe_2_C particles, the GMX-MFe samples exhibit optimized and tunable permittivity in 2–18 GHz. Obvious resonance peaks appeared in the *ε''*-*f* curves of the GMX-MFe samples and the initial value of *ε''* is reduced compared to GMX-M3, which indicates the improvement of polarization loss and reduction of conductive loss (Fig. 7b, h).

The mechanism of the above phenomenon in GMX-MFe samples can be summarized as follows: 1) The introduction of Fe_2_C particles optimizes the capacity of impedance matching by breaking the conductive network and serving as barriers to restrict the movement of charges. 2) The heterointerfaces among MXene, rGO, TiO_2_, and Fe_2_C promote multiple interfacial polarization [17]. 3) The abundant polar functional groups in MXene and rGO provide intense dipole polarization [9]. 4) The defects induced by the introduction of Fe_2_C and in-situ generated TiO_2_ particles jointly contribute to defect-induced dipole polarization [72]. Therefore, the dielectric loss mechanism in the samples can be attributed to the coupling of space charge polarization, dipole polarization and conductive loss [73].

All GMX-MFe samples share the same trend of complex permittivity curves and multi-polarization coupling mechanisms owing to similar components and microstructures. However, under the effect of different MXene/rGO intercalation, the average values of complex permittivity and magnitude of resonant peak first increase and then decrease from GMX-MFe1 to GMX-MFe4, reaching the maximum value in GMX-MFe3. According to the above microstructure analysis and MGH model, the intercalation degree of MXene and rGO nanosheets in the GMX-MFe samples should be GMX-MFe3 > GMX-MFe4 > GMX-MFe2 > GMX-MFe1. The higher the intercalation degree of MXene and rGO nanosheets, the higher the polarization interface area and polarization charge density. Therefore, GMX-MFe3 with alternated intercalation units has the most significant polarization, which is highly consistent with our simulation results in Fig. 6.

The *μ'* and *μ''* curves of different samples are shown in Fig. 7c–d. Since GMX3 and GMX-M3 are not magnetic, the *μ'* and *μ''* values are around 1 and 0, respectively. The introduction of Fe_2_C particles leads to enhanced magnetic loss in the GMX-MFe samples (Fig. 6e). The fluctuations of *μ'* and *μ"* values on the curve are mainly caused by the natural and exchange resonances (Fig. S17) [27]. Moreover, the saturation magnetization increases with MXene ratio (Fig. S9b) due to more ferrocene converted to Fe_2_C originated from the rapid temperature rise induced by MXene [45]. When complex dimensional gradient structures with large interfacial polarization are exposed to an alternating electromagnetic field, a current is generated, resulting in a magnetic field opposite to the external one. Such opposite induced field will cause fluctuations in magnetic field energy, contributing to negative values of *μ"* (Fig. 7d) [46, 74–76].

In addition, the values of $\tan \delta_{E}$ for all the magnetic microspheres are higher than those of $\tan \delta_{M}$, indicating that dielectric loss is the main EMW loss mechanism in GMX-MFe samples. Furthermore, the Cole–Cole curves can be obtained from the following relation according to the Debye theory [30, 46]:

5 $$ \left( {\varepsilon^{\prime} - \frac{{\varepsilon_{s} + \varepsilon_{\infty } }}{2}} \right)^{2} + \left( {\varepsilon^{\prime\prime}} \right)^{2} = \left( {\frac{{\varepsilon_{s} - \varepsilon_{\infty } }}{2}} \right)^{2} $$

where *ε*_*s*_ is the static dielectric constant and *ε*_*∞*_ represents the dielectric constant in the high-frequency limit. Generally speaking, the number of the semicircle part and the length/slope of the linear part in the *ε'-ε"* curve reflect the Debye relaxation process and conductive loss, respectively. From GMX3 to GMX-MFe, the huge increase in heterointerfaces greatly reinforces the polarization effect. The further increase of multiple semicircles in the curve shows a synergistic effect between multiple polarization relaxations (Fig. 7g). The above phenomena indicate that the adjustment of the heterointerface can enhance the multiple polarization and boost the EMW absorption, which can be explained by the MGH model. Due to the unique crystal and electronic structures present in the samples, charge separation and charge transfer would occur near the interface region under the action of alternating electromagnetic fields [10, 17]. These polarized charges eventually accumulate at the heterointerfaces to form micro-capacitance structures, which triggers the relaxation of the built-in electric fields.

Besides, abundant functional groups and defects within the heterostructures contribute to electric dipole polarization [77]. The slope and length of the linear part of the curve first increase and then decrease, demonstrating that the conductive loss is regulated [20]. To further confirm the attenuation mechanism, the polarization (*ε*_*p*_*′′*) and conduction (*ε*_*c*_*′′*) contributions to the dielectric loss have been calculated based on the Debye theory [7, 78] according to Eq. (6):

6 $$ \varepsilon^{\prime\prime}(\omega ) = \varepsilon^{\prime\prime}_{P} + \varepsilon^{\prime\prime}_{c} = (\varepsilon_{s} - \varepsilon_{\infty } )\frac{\omega \tau }{{1 + \omega^{2} \tau^{2} }} + \frac{\sigma }{{\varepsilon_{0} \omega }} $$

where *σ*, *ω* are the electrical conductivity and angular frequency, respectively. It can be concluded that the construction of 2D/2D/0D/0D heterostructures significantly increases the polarization loss (Fig. 7h). Additionally, through heterostructure modulation, the samples exhibit a high ratio of polarization loss, exceeding 70% of the overall dielectric loss. This result demonstrates the advantages of customizing hierarchical architectures via heterointerface engineering and composition control to strengthen polarization effects (Table S1).

To demonstrate the effect of heterostructure adjustment on the EMA performance, the RL values are calculated from the complex permittivity (*ε*_*r*_) and the complex permeability (*μ*_*r*_) based on the transmission line theory by the following Eqs. (7−8):

7 $$  Z_{{in}}  = Z_{0} \sqrt {\frac{{\mu _{r} }}{{\varepsilon _{r} }}} \tanh \left( {j\frac{{2\pi fd\sqrt {\mu _{r} \varepsilon _{r} } }}{c}} \right)  $$

8 $$ RL = 20\log_{10} \left| {{{\left( {Z_{in} - Z_{0} } \right)} \mathord{\left/ {\vphantom {{\left( {Z_{in} - Z_{0} } \right)} {\left( {Z_{in} + Z_{0} } \right)}}} \right. \kern-0pt} {\left( {Z_{in} + Z_{0} } \right)}}} \right| $$

where *Z*_*in*_ is the normalized input impedance of the absorber, *Z*_*0*_ is the impedance in free space, *f* represents the frequency of EMW, *d* is the thickness of the sample and *c* corresponds to the speed of light. Microwave absorption performance can be evaluated in terms of *RL*_min_ and effective absorption bandwidth (EAB, frequency range where RL ≤  − 10 dB) [79].

**Fig. 8:**
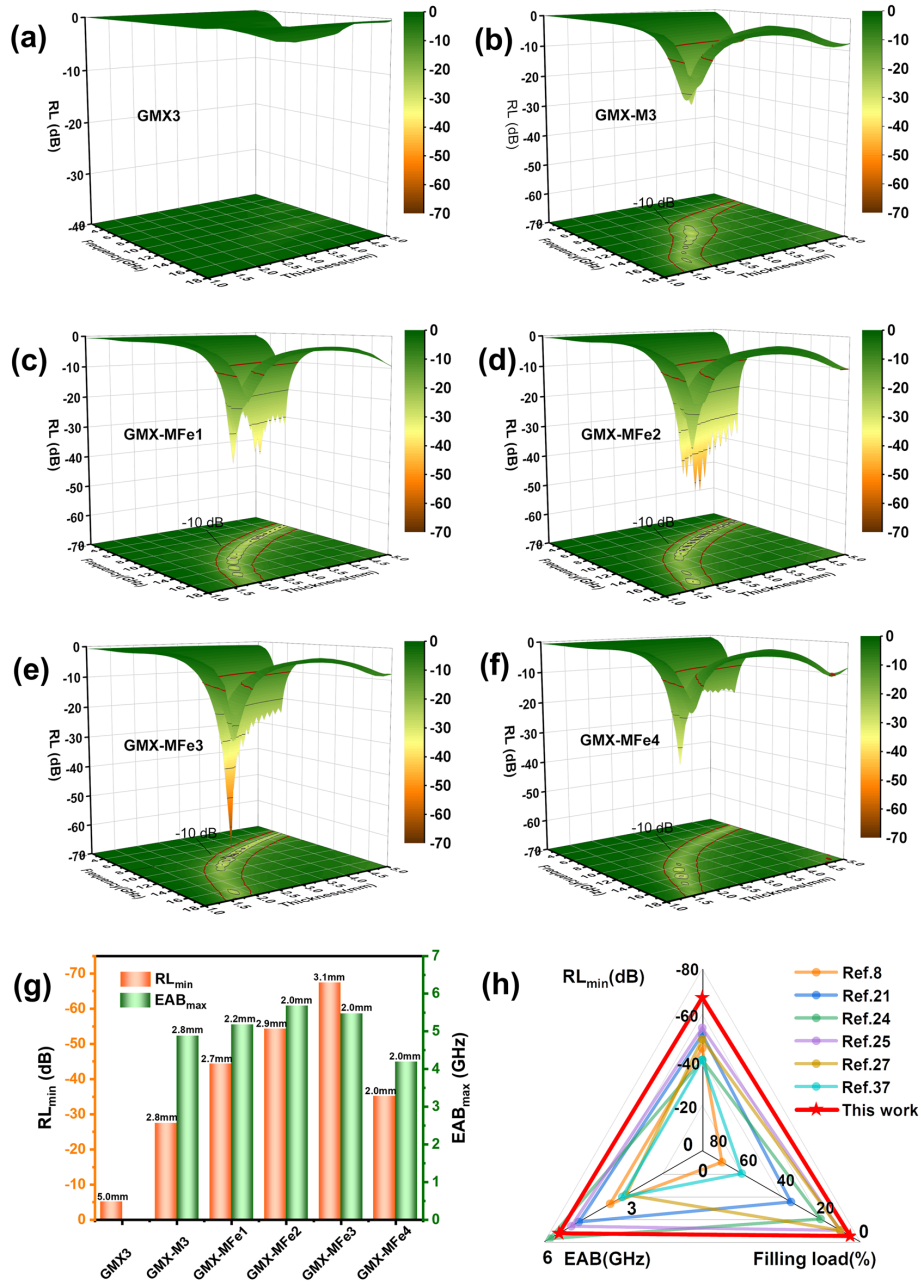
3D RL plots of **a** GMX3, **b** GMX-M3, **c** GMX-MFe1, **d** GMX-MFe2, **e** GMX-MFe3, and **f** GMX-MFe4. **g** Comparison of *RL*_min_ and *EAB*_max_ values and corresponding thicknesses for all the samples. **h** Radar comparison chart of EMA performances for related 3D graphene or/and MXene based absorbers compared with this work

Figures 8 and S18 show the RL values for different samples at various thicknesses in the frequency range of 2–18 GHz. The absorption peaks move towards lower frequency as the tested thickness increases, which is in accordance with the quarter-wavelength (*λ*/4) cancellation theory (Fig. S19) [80]. Since the insulating and weakly polarized GO nanosheets are intercalated between single or few layers of MXene nanosheets [6], a simple structure is formed. Consequently, the EMA performance of GMX3 is poor (Fig. 8a). After microwave irradiation, GO and MXene undergo redox reactions, and 2D/2D/0D (rGO/MXene/TiO_2_) heterojunctions are introduced into GMX-M samples. The microwave attenuation is thus improved by polarization and conductive losses in the multi-level heterostructure of GMX-M3, showing *RL*_min_ and EAB values of −27.4 dB and 4.88 GHz (thickness of 2.8 mm) (Fig. 8b), respectively.

With the introduction of Fe_2_C, the synergistic effect between magnetic and dielectric losses is promoted while fully exploiting the interface and further increasing the interfacial charge accumulation [8]. As a result, magnetic GMX-MFe exhibits superior EMA performance (Figs. 8e and S18). In particular, GMX-MFe2 and GMX-MFe3 display optimal *RL*_min_ values of −54.3 dB (2.9 mm thickness), and −67.4 dB (3.1 mm thickness) and EAB_max_ values of 5.68 and 5.47 GHz (2.0 mm thickness), respectively. Furthermore, when the absorber thickness is adjusted (1.0–5.0 mm), the effective absorption peak of GMX-MFe3 shifts from 4.32 to 18 GHz, covering most of the C-band and all of the X and Ku-bands (Fig. S18c). An overall comparison of all the samples demonstrates that GMX-MFe3 possesses both optimal *RL*_min_ value and relatively broad *EAB*_max_, and thus the best EMA performance (Fig. 8g). These hierarchical heterostructures generate abundant polarization sites while providing a high density of polarization charges, triggering strong polarization and ultimately leading to optimized EMA performance. Therefore, the construction of compositionally controllable heterointerfaces by the rational design of intercalated heterostructures is an effective strategy to improve EMA performance.

Compared with previously reported hierarchical structures prepared by conventional methods (Table S2), spray-freeze-drying combined with microwave irradiation can maximize the interfacial area and polarized charge density through precise multi-layer assembly, multi-scale components and porous skeleton structure. Subsequently, the electromagnetic parameters and EMA properties could be tailored through the modulation of heterointerfaces. Moreover, the employed microwave-assisted synthesis is much more efficient and energy-saving than standard heating, avoiding nanoparticle agglomeration. This enabled an excellent EMA performance with a filler loading of 5 wt%, which is lower than most of the reported absorbers (Fig. 8h).

It is known that excellent impedance matching and attenuation capability are important factors in determining the EMA performance. The attenuation capability can be assessed by the attenuation constant *α* [81]:

9 $$ \alpha = \frac{\sqrt 2 \pi f}{c} \times \sqrt {\left( {\varepsilon^{\prime\prime}\mu^{\prime\prime} - \varepsilon^{\prime}\mu^{\prime}} \right) + \sqrt {\left( {\varepsilon^{\prime\prime}\mu^{\prime\prime} - \varepsilon^{\prime}\mu^{\prime}} \right)^{2} - \left( {\varepsilon^{\prime\prime}\mu^{\prime} - \varepsilon^{\prime}\mu^{\prime\prime}} \right)^{2} } } $$

**Fig. 9:**
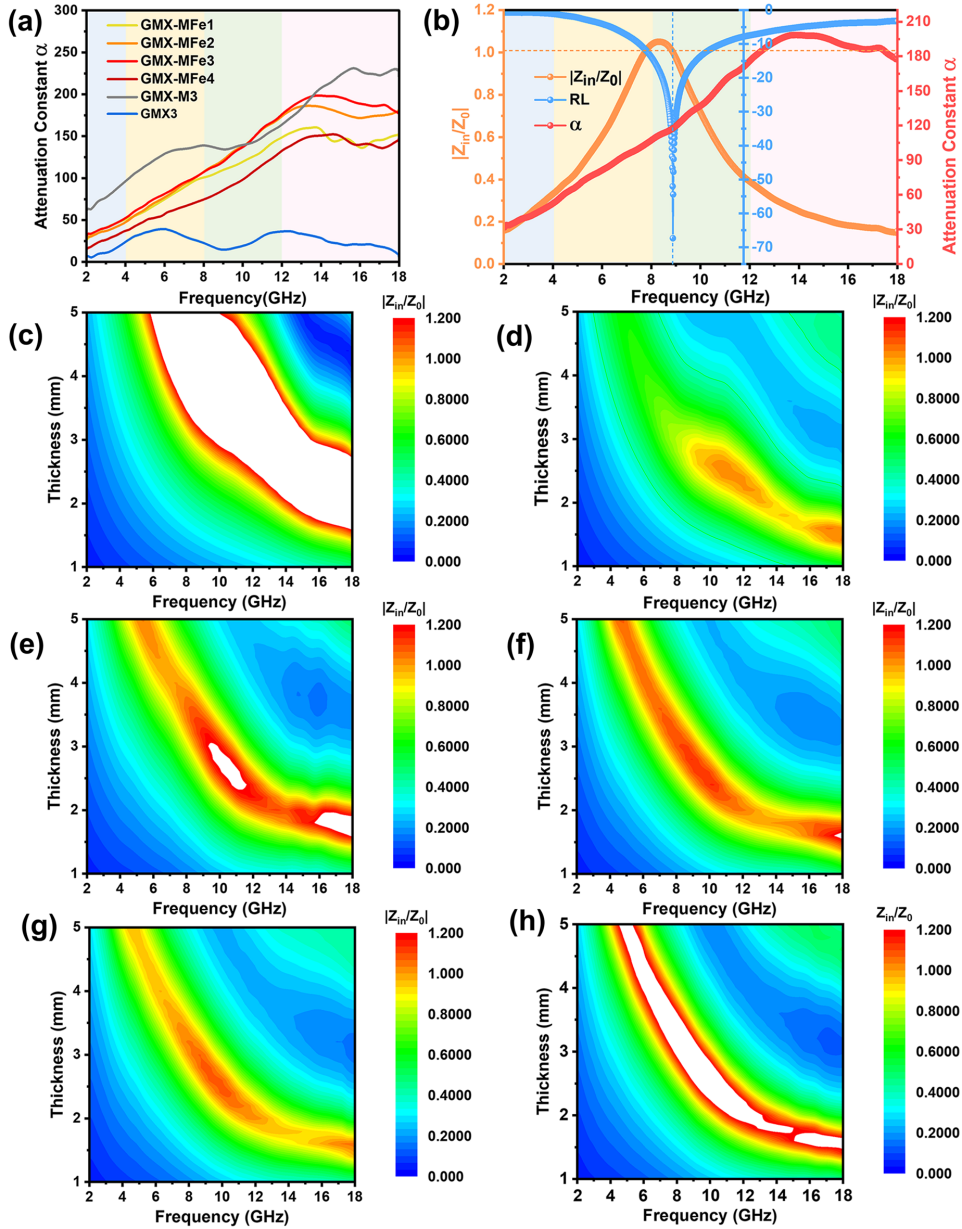
**a** Attenuation constant *α* of different samples. **b** Frequency-dependent RL, |*Z*_*in*_/*Z*_*0*_|, and *α* values for GMX-MFe3. 2D contour maps of |*Z*_*in*_/*Z*_*0*_| for **c** GMX3, **d** GMX-M3, **e** GMX-MFe1, **f** GMX-MFe2, **g** GMX-MFe3 and **h** GMX-MFe4

As shown in Fig. 9a, the *α* value of the sample increases significantly after microwave irradiation indicating that the EMW attenuation ability is enhanced. Besides, the effect of impedance matching needs to be considered. The impedance matching characteristics are usually reflected in the value of |*Z*_*in*_/*Z*_*0*_| in which a value closest to 1 represents the best impedance matching [82]. Figure 9c–h show the |*Z*_*in*_/*Z*_*0*_*|* 2D contour plots for different samples. The plot of GMX-MFe3 presents the largest area with |*Z*_*in*_/*Z*_*0*_| ratio close to 1 (the orange region) and thus the best impedance matching. By constructing 2D/2D/0D/0D intercalated heterostructures containing magnetic Fe_2_C nanoparticles in porous GMX-MFe and adjusting the intercalation periodicity to modulate the phase composition of the interface, the tuning of electromagnetic parameters and the improvement of impedance matching are ultimately achieved. The optimized impedance matching and multi-effective synergistic loss mechanism endow GMX-MFe3 with excellent EMA performance. Moreover, Fig. S20 reflects the relationship between *RL*_min_, *α* and *Z* values, which also demonstrates that the combination of appropriate attenuation loss and ideal impedance matching facilitates optimal EMA performance.

**Fig. 10:**
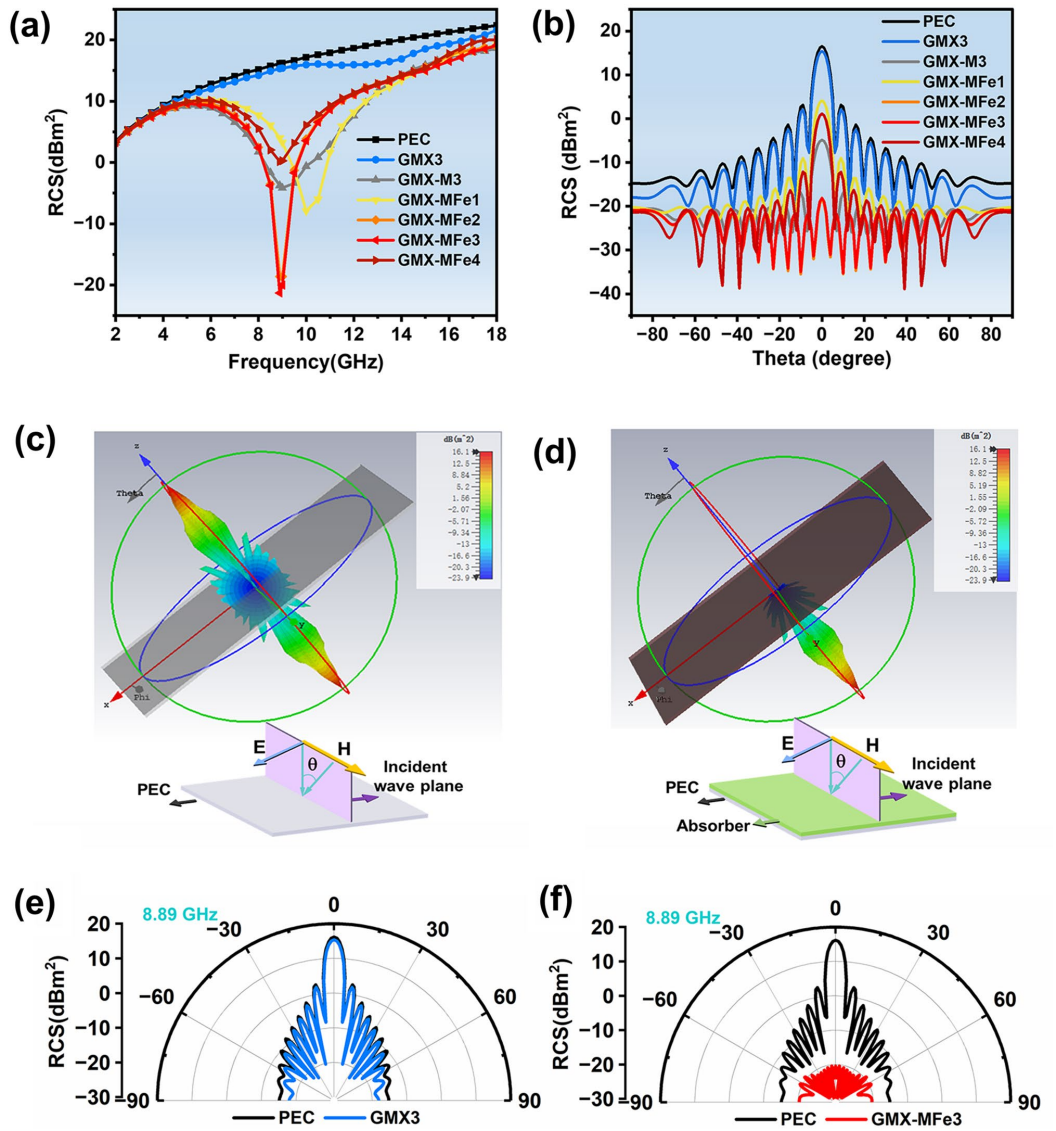
**a** Broadband RCS results. **b** RCS simulated curves of composites containing pure PEC and PEC coated with an absorbing layer. 3D radar wave scattering signals of **c** PEC and **d** GMX-MFe3. RCS in polar coordinate system of **e** GMX-3 and **f** GMX-MFe3 coated PEC plate at 8.89 GHz

To evaluate the EMA performance of the samples for practical applications, the RCS values (Figs. 10 and S21) of the PEC plate and absorber-coated PEC plates were calculated using CST Studio Suite (Section S13). From Fig. 10a, the RCS values of GMX-M3 and GMX-MFe series coated PEC plates are significantly reduced compared to the pure PEC plate under vertical incident EMW (*θ* = 0°). Moreover, the optimal RCS reduction value (34.66 dBm^2^) is achieved in the GMX-MFe3 at around 8.89 GHz. To further evaluate the RCS reduction capacity in all directions, the 3D and 2D radar wave scattering signals at 8.89 GHz are shown in Fig. 10e–f. It is worth noting that the RCS values of GMX-M3 and GMX-MFe series coated PEC plates are shrinking from − 90° to 90°, which implies a reduction mechanism of RCS based on absorption rather than multi-directional scattering. The RCS value of GMX-MFe3 is close to −20 dB m^2^ (large EMA capacity) from − 90° to 90°, which agrees with the EMA performance summarized in Fig. 8 [83]. By comparing the RCS values of GMX-MFe3 with GMX3 at different frequency points in the X band (Fig. S23), it can be concluded that the rational construction of hierarchical heterostructures can effectively reduce the RCS values. The above results show a promising application of magnetic layered porous microspheres as lightweight EMA coatings.

**Fig. 11:**
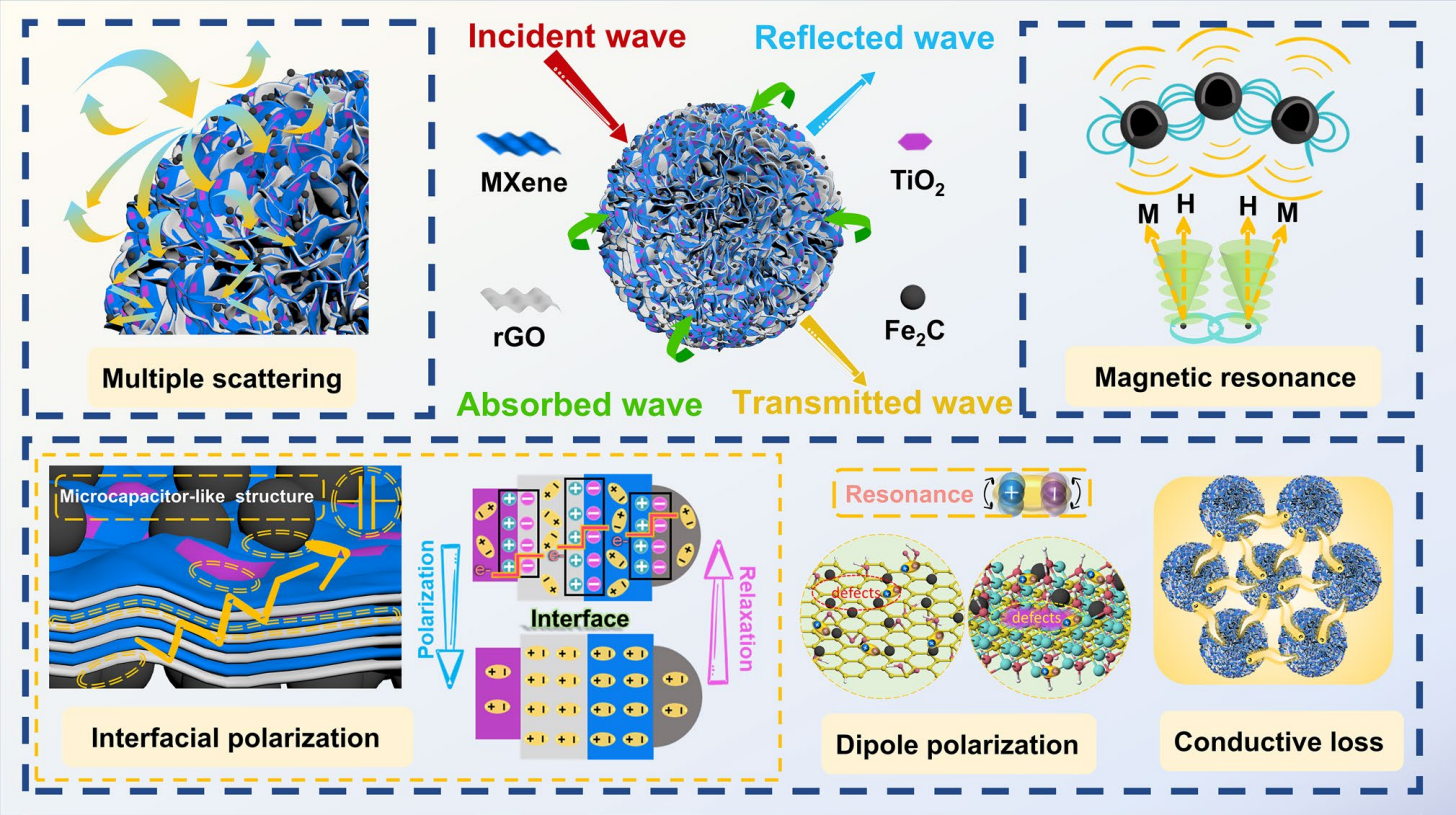
Schematic illustration of electromagnetic wave absorption mechanism of GMX-MFe microspheres

Based on the above analyses, Fig. 11 shows the microwave absorption mechanism of the 3D rGO/MXene/TiO_2_/Fe_2_C hierarchically structured microspheres. Heterointerface engineering via heterostructure (rGO, MXene, TiO_2_, and Fe_2_C), multi-scale components (2D rGO/MXene, 0D TiO_2_/Fe_2_C), periodic multi-layer assembly and porous skeleton results in optimized impedance matching and facilitated EMW dissipation. Firstly, multi-polarization plays an important role in dielectric loss. The 2D/2D/0D/0D multi-level heterostructures introduce abundant interfaces between rGO, MXene, TiO_2_ and Fe_2_C. Carriers then gather at these interfaces and arrange themselves to form numerous heterojunction micro-capacitors, which eventually trigger interfacial polarization [10, 25]. Secondly, plenty of defects and functional groups contribute to dipole polarization and thus to dielectric loss. Next, the 3D porous conductive networks formed by the heterogeneous structural units not only increase the conductive loss, but also extend the EMW propagation paths to achieve multiple scattering, further attenuating the microwave energy. Finally, magnetic Fe_2_C uniformly dispersed on the surface of the heterostructure contributes to magnetic and dielectric loss synergism through the resonance effect, thus providing the 3D rGO/MXene/TiO_2_/Fe_2_C microspheres with excellent EMA performance.

## Conclusions

3D porous rGO/MXene/TiO_2_/Fe_2_C hierarchical microspheres have been successfully designed and prepared based on heterointerface engineering via simple and versatile spray-freeze-drying followed by microwave irradiation. Such heterointerfaces were engineered at the micro- and nanoscales through GO/MXene mass ratio and in-situ thermal reduction of GO, MXene oxidation and ferrocene decomposition. This promoted multiple losses, impedance matching and scattering in the porous structures. With merely 5 wt% filler loading, the GMX-MFe3 achieved a *RL*_min_ value of − 67.4 dB, EAB of 5.47 GHz at a thickness of 2.0 mm. Moreover, the RCS attenuation value can reach 34.66 dB m^2^. Overall, this work adopts a rational micro-nano structural design to customize EMA, effectively integrating functional units of different dimensions and multiple loss mechanisms to fully exploit interfacial effects. Both experimental and simulation results demonstrate the advantages of tailoring hierarchical architectures in improving polarization effects based on heterointerface engineering, which is pivotal for developing high-performance EMA materials. At the same time, these outcomes stimulate the controlled assembly of 2D materials and their applications in the fields of electromagnetic protection and stealth technology.

### Supplementary Information

Below is the link to the electronic supplementary material.

Supplementary file1 (MP4 7301 kb)

Supplementary file2 (PDF 6929 kb)
