# Supplementary material for: Boosting Interfacial Polarization Through Heterointerface Engineering in MXene/Graphene Intercalated-Based Microspheres for Electromagnetic Wave Absorption
Source: Nanomicro Lett. 2023 Jun 7;15:152. doi: 10.1007/s40820-023-01123-4 (PMC10247949; doi:10.1007/s40820-023-01123-4)
Supplement: Supplementary file 2 — Supplementary file2 (PDF 6929 kb) [file 40820_2023_1123_MOESM2_ESM.pdf]

Supplementary Information for

Boosting Interfacial Polarization through Heterointerface Engineering in MXene/Graphene Intercalated-Based Microspheres for Electromagnetic Wave Absorption

Ge Wang[1, #], Changfeng Li[1, #], Diana Estevez[1, 2], Peng Xu [1, 3, *], Mengyue Peng[1], Huijie Wei[1], Faxiang Qin[1, *]

[1]Institute for Composites Science Innovation (InCSI), School of Materials Science and Engineering, Zhejiang University, 38 Zheda Road, Hangzhou 310027, P. R. China

[2]Ningbo Institute of Technology, Zhejiang University, 1 Qianhu South Rd, Ningbo 315100, P. R. China

[3]Foshan (Southern China) Institute for New Materials, Foshan, P. R. China

[#]Ge Wang and Changfeng Li contributed equally to this work.

*Corresponding authors. E-mail: pengxu@zju.edu.cn (Peng Xu), faxiangqin@zju.edu.cn (Faxiang Qin)

S1 Interfacial Polarization Model Analysis

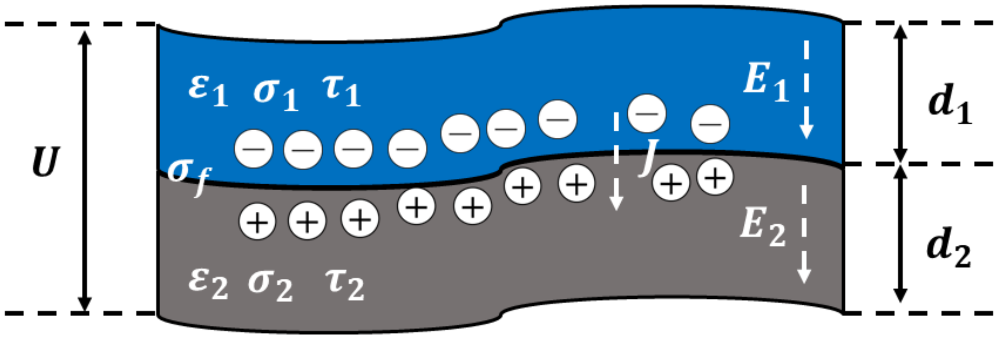

Fig. S1 Schematic diagram of interfacial polarization

In this section, the Maxwell-Wagner interfacial polarization model will be described in detail[S1, S2]. The relaxation time τ is defined as the carrier migration time in a dielectric with permittivity of ε and conductivity of σ under the action of electric field:

$$\tau = \frac{\varepsilon}{\sigma} \tag{S1.1}$$

As shown in Fig. S1, when the electromagnetic wave (EMW) is applied to a double-layer dielectric, the electric field part of Maxwell equations can be shown as follows:

$$\nabla \cdot \vec{D} = \rho_s$$

$$\nabla \times \vec{E} = -\frac{\partial \vec{B}}{\partial t}$$

$$\vec{D} = \varepsilon \vec{E}$$

$$\vec{j} = \sigma\vec{E} \tag{S1.2}$$

According to Equations (S1.2), when the two phases have different dielectric properties (ε, σ), polarization charge accumulation will occur at the heterointerface due to different relaxation times:

$$\nabla \cdot \vec{D} = \nabla \cdot \varepsilon\vec{E} = \nabla \cdot \left(\frac{\varepsilon}{\sigma}\right)\vec{j} = \nabla \cdot \tau\vec{j} = \rho_s$$

$$(\tau_1 - \tau_2)\vec{j} = \sigma_f \tag{S1.3}$$

where σ_f is the accumulated charge at the interface, τ_1 and τ_2 is the relaxation time of two phases, respectively.

Under a step electric field of modulo U, according to Eq. (S1.2), at the interface:

$$D_1 = \varepsilon_1 E_1 = D_2 = \varepsilon_2 E_2 \tag{S1.4}$$

$$U = E_1 d_1 + E_2 d_2 \tag{S1.5}$$

where D_1 and D_2 are the electric displacements of the two phases, respectively. ε_1 and ε_2 represent the permittivity of the two phases, respectively. E_1 and E_2 denote the electric applied to two phases, respectively. d_1 and d_2 stands the thickness of two phases, respectively.

The electric field strength can be obtained according to Eqs. (S1.4) and (S1.5):

$$E_1 = \frac{\varepsilon_2 U}{(\varepsilon_1 d_2 + \varepsilon_2 d_1)} \tag{S1.6}$$

$$E_2 = \frac{\varepsilon_1 U}{(\varepsilon_1 d_2 + \varepsilon_2 d_1)} \tag{S1.7}$$

The charge accumulated at the interface is the integral of current density over time [S1]:

$$\sigma_f = \int [J_1 - J_2]\, dt = \frac{\varepsilon_2\sigma_1 - \varepsilon_1\sigma_2}{\sigma_1 d_2 + \sigma_2 d_1} \cdot U \tag{S1.8}$$

S2 Synthesis of GO and MXene

Figure S2(a) shows the preparation of graphene oxide (GO) by the modified Hummers' method, where graphite flakes (Fig. S2(c)) were intercalated and oxidized using potassium permanganate and concentrated sulfuric acid. The obtained graphite oxide was then exfoliated by ultrasonication to prepare the aqueous dispersion of GO nanosheets. Fig. S2(d-e) shows the successful exfoliation of GO nanosheets. Furthermore, the synthesis process of MXene nanosheets is shown in Fig. S2(b). The aluminum atomic layers in the Ti_3AlC_2 precursor (MAX phase) were selectively removed as reported [S3]. The obtained multilayer MXene can be exfoliated into MXene nanosheets after ultrasonication (Fig. S2(g-h)).

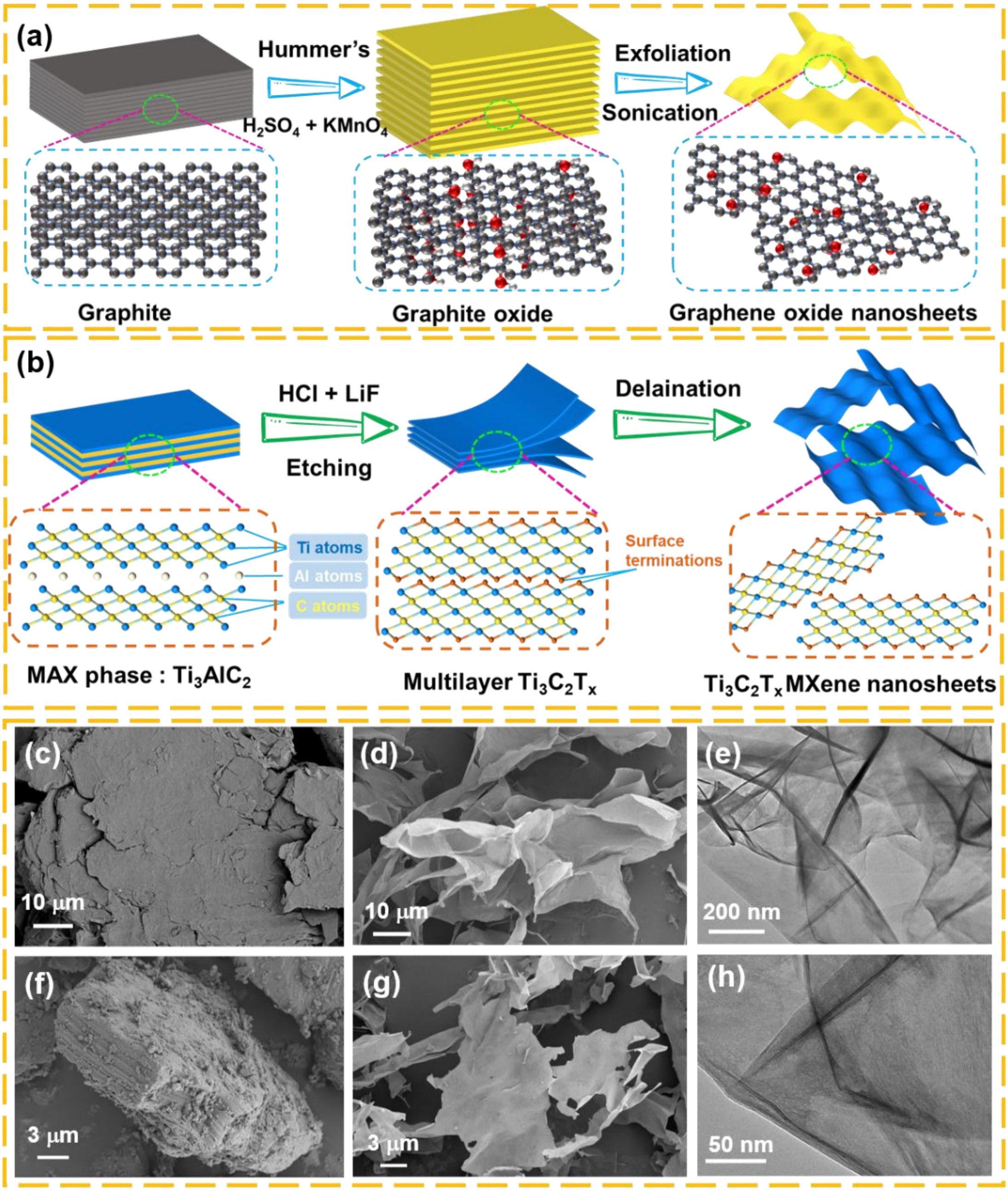

Fig. S2 Schematic diagram illustrating the fabrication of (**a**) graphene oxide nanosheets and (**b**) $Ti_3C_2T_x$ MXene nanosheets. SEM images of (**c**) graphite, (**d**) graphene oxide nanosheets, (**f**) Ti_3AlC_2 (MAX phase) and (**g**) $Ti_3C_2T_x$ MXene nanosheets. TEM images of (**e**) graphene oxide nanosheets and (**h**) MXene nanosheets

S3 Topography of GO and MXene Nanosheets

The thickness of the ultra-thin GO monolayer nanosheets is about 1.2 nm. The nanosheets exhibit a wrinkle structure (represented by the green dashed lines in Fig. S3(a)). The thickness of the monolayer MXene nanosheets is about 1.5 nm. Therefore, both nanosheets exhibit ultrathin lamellar structures.

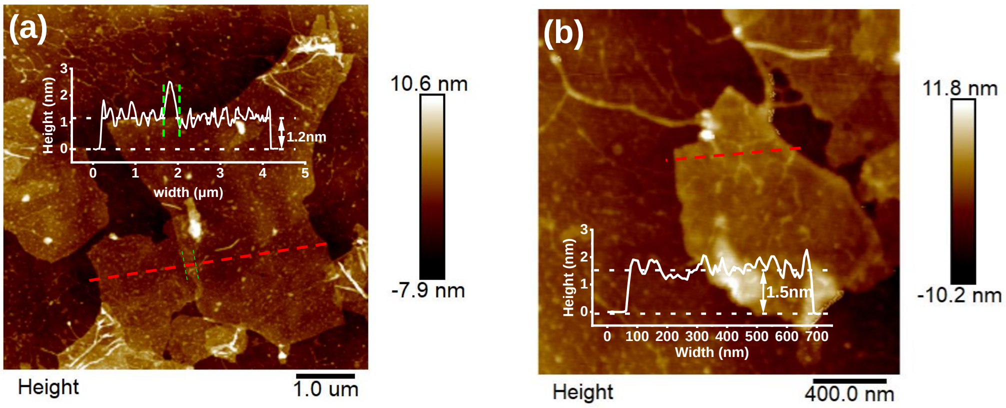

Fig. S3 2D surface topography of (**a**) GO and (**b**) MXene monolayers

S4 Homogeneity of GO and MXene Dispersions

Due to the interaction between adjacent thin nanosheets, homogeneous dispersions can be formed by GO, MXene, and a mixture of both nanosheets (Fig. S4(a-c)), respectively. In particular, the dispersion of GMX3 in water by stirring also forms stable homogeneous solutions, which can be confirmed by the Tyndall scattering effect (Fig. S4(d)).

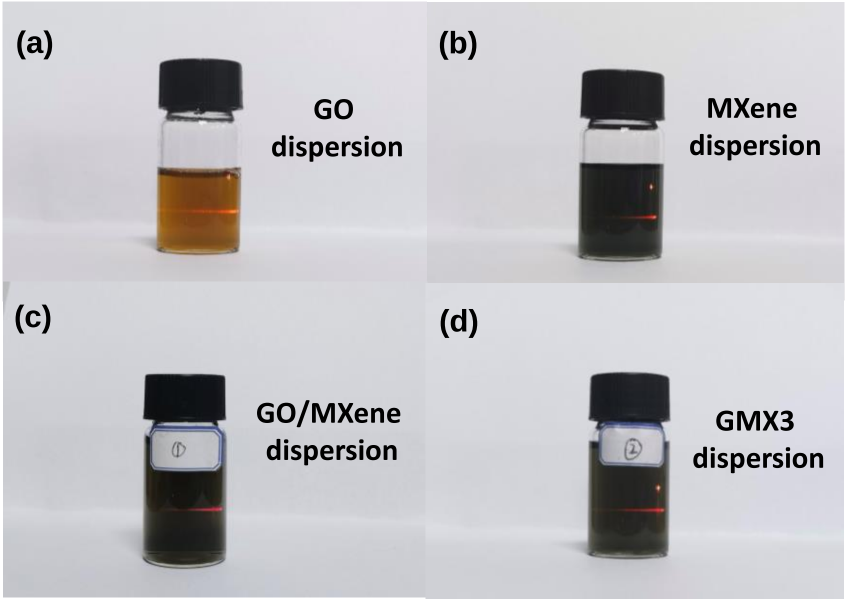

Fig. S4 Photographs of the (**a**) GO suspension, (**b**) MXene suspension, (**c**) GO and MXene hybrid suspension with mass ratios of 1:1 and (**d**) stir-treated GMX3 in water dispersion

S5 Structural and Elemental Analysis

The size of the obtained GOS is significantly larger than that of MXS due to the difference in the gelation ability of GO and MXene (Fig. S5). In addition, GOS showed structural units that tended to be stacked (shown as ‘petals’ with wrinkles that tend to be closed), while MXS showed straighter ‘petals’ and looser structures (shown as microsphere structures with cracks). The above problems can be largely solved by assembling GO and MXene into sandwich microspheres by spray-freeze-drying method.

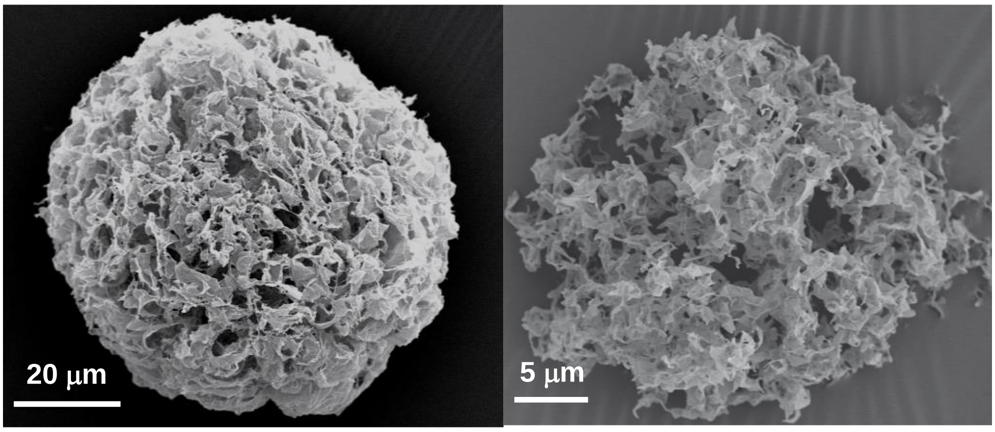

Fig. S5 SEM images of (**a**) GOS and (**b**) MXS

As shown in Fig. S6, the nanoparticles are uniformly distributed on the lamellar structural units of the microspheres without severe agglomeration. It is worth mentioning that the distribution of spherical nanoparticles in Fig. S6(c) coincides with the distribution of Fe in the energy dispersive spectra (EDS) of Fig. S6(d), indicating that Fe_2C and TiO_2 exhibit spherical and elongated morphologies, respectively.

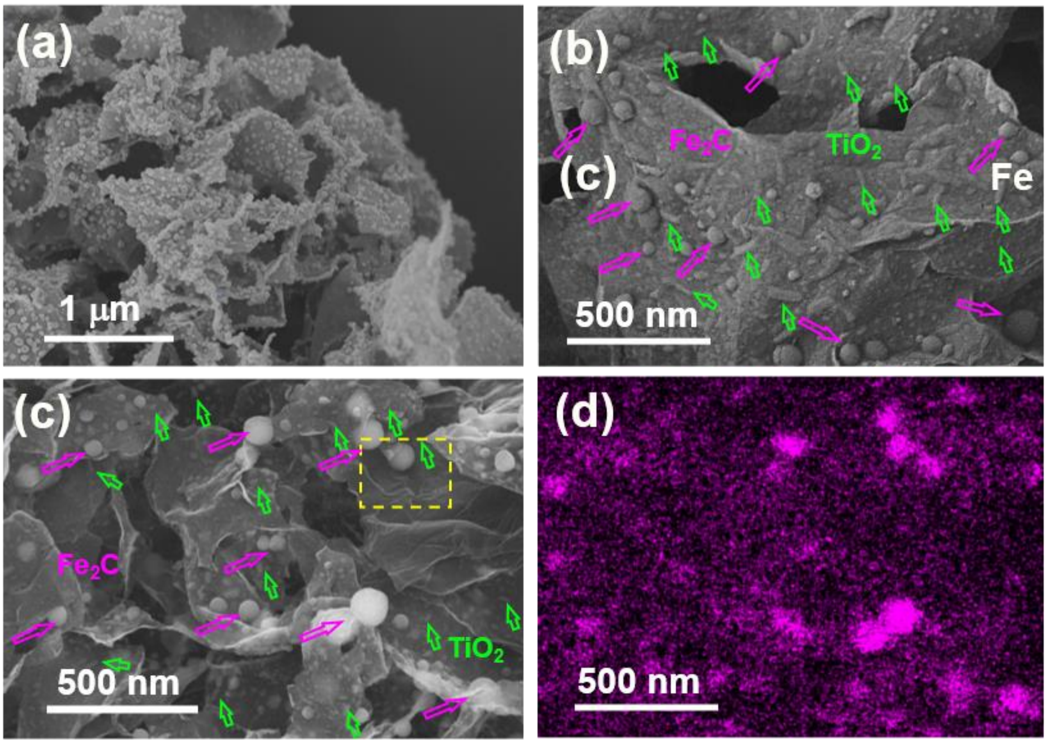

Fig. S6 (**a-c**) High magnification SEM images of GMX-MFe3. (**d**) Fe element mapping image

As shown in Fig. S7(a), for the GO spheres (GOS), when the droplets were rapidly frozen by liquid nitrogen, the diffraction peak does not appear at ~10 ° due to the random distribution of GO, but a broad diffraction peak appears at ~21 °, and the diffraction peak of GOS-M obtained after microwave-assisted treatment does not change significantly. The (001) diffraction peak of GO can be observed at 10.1 ° when GOS is dispersed in water to obtain GOS-M. As a comparison, GO aerogels were obtained using the freeze-drying method with the same precursor solution concentration, which shows the (001) characteristic peak at 9.46 °. The GO aerogel-M obtained by

microwave treatment shows the peak of rGO at 25 °. This indicates that spray-freeze-drying plays an important role in preventing random self-stacking of the nanosheets when they are assembled and that microwave irradiation can reduce GO. Furthermore, in Fig. S7(b), the (002) crystal plane shifts to a lower angle and the (104) crystal plane disappears due to the chemical etching of Ti_3AlC_2, indicating the successful removal of the Al layer and the delamination of $Ti_3C_2T_x$. The diffraction peaks of the obtained MXS-M show no obvious changes, and no diffraction peaks of TiO_2 appeared, indicating that MXene was not significantly oxidized after microwave irradiation.

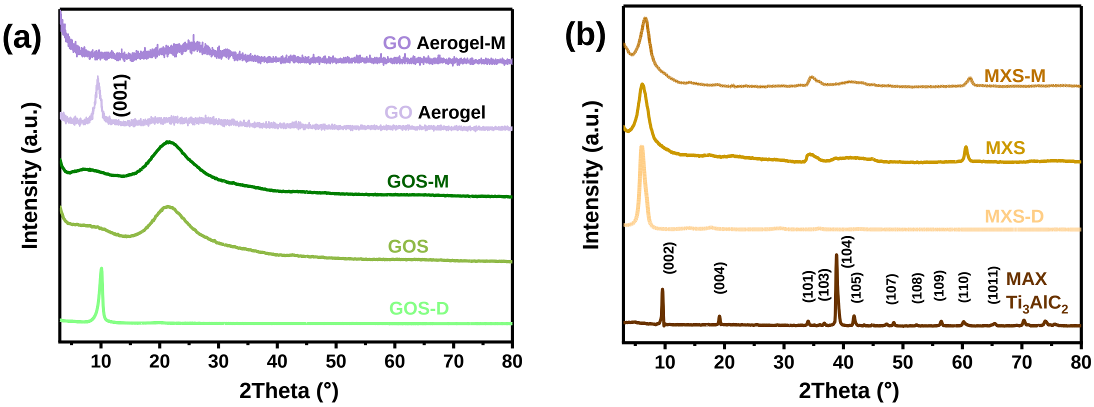

Fig. S7 (**a**) XRD patterns of GO Aerogel-M, GO Aerogel, GOS-M, GOS and GOS-D, (**b**) XRD patterns of Ti_3AlC_2, MXS-D, MXS and MXS-M

As shown in Fig. S8, a superposition peak appears in the XRD pattern of GMX3-D, which consists of the diffraction peak of MXene (002) crystal plane at 7.96° and the diffraction peak of GO (001) crystal plane at 8.95°. This is due to the redispersion process of GMX3 microspheres that disrupts the GO/MXene intercalation structures, resulting in the restacking of GO and MXene This also corroborates the important role of the spray-freeze-drying method in the formation of GO/MXene intercalation layers. In addition, the intensity of the diffraction peak of MXene (002) crystal plane in GMX3 is significantly lower than that of GMX3-D, which is due to the separation of MXene nanosheets by GO nanosheets in the intercalated structure.

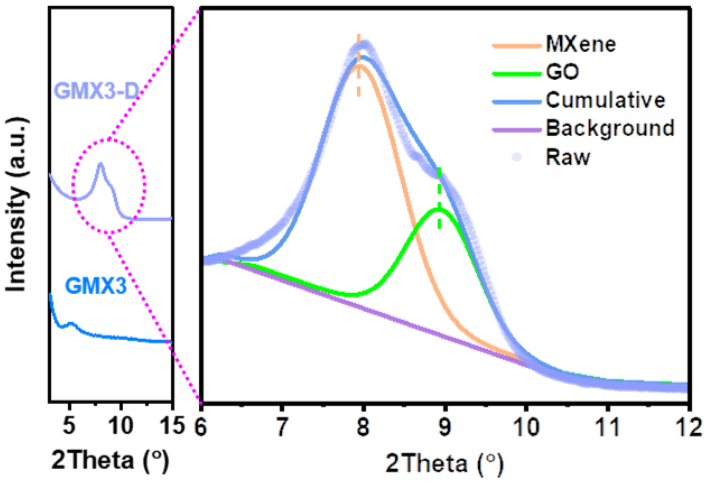

Fig. S8 XRD patterns of GMX3-D with partial high magnification

After microwave irradiation, the GMX-MFe microspheres showed similar diffraction peaks, proving the presence of TiO_2 and Fe_2C. In addition, the saturation magnetization intensity increased with the increase of GO to MXene ratio (Fig. S9(b)), which may originate from the rapid temperature rise caused by MXene. The intense heat generated when MXene was subjected to microwave irradiation would lead to more ferrocene conversion to Fe_2C.

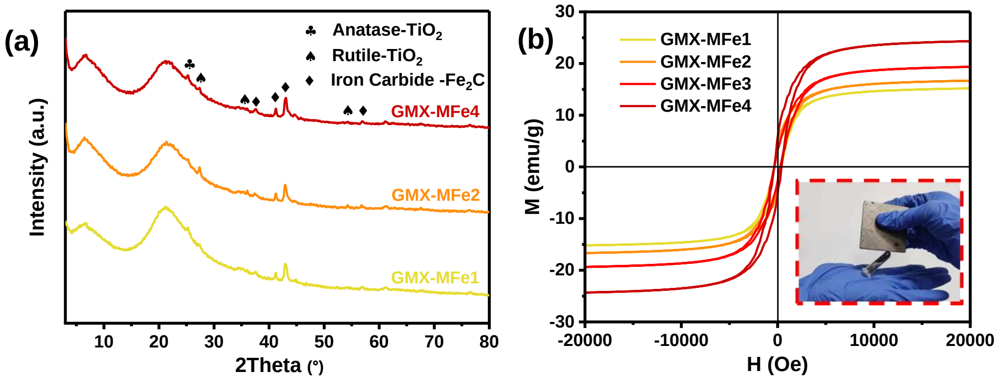

Fig. S9 (a) XRD patterns of GMX-MFe1, GMX-MFe2 and GMX-MFe4, (**b**) Magnetic property of GMX-MFe1, GMX-MFe2, GMX-MFe3 and GMX-MFe4 measured at room temperature

After microwave irradiation, the oxygen-containing functional groups of GMX-M and GMX-MFe are reduced. For GOS and MXS, deoxygenation of the former occurred after microwave irradiation, while the latter was not oxidized.

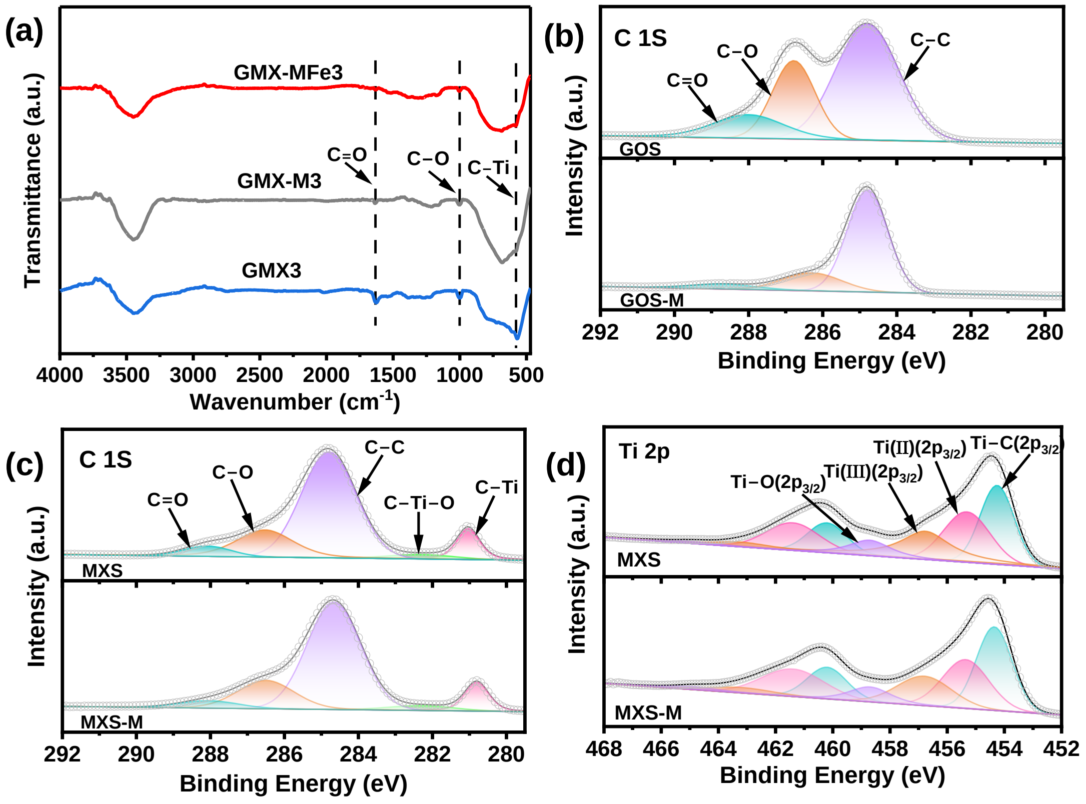

Fig. S10 (**a**) FTIR spectra of GMX3, GMX-M3 and GMX-MFe3, (**b**) high-resolution XPS spectra C 1s of GOS and GOS-M. (**c**) C 1s and (**d**) Ti 2p of MXS and MXS-M

HRTEM images show that the lamellar spacing of MXene in the structural units of MXM is 1.46 nm.

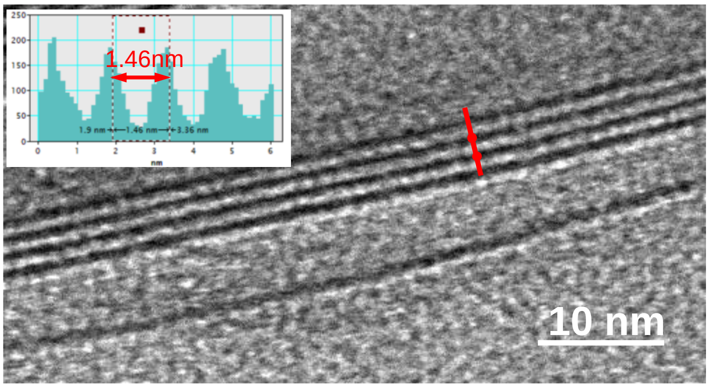

Fig. S11 HRTEM images of MXS, and the layer spacing of MXene along the red line (inset)

Based on the SEM images in Fig. S12, it can be found that two types of nanoparticles, spherical and elongated, are anchored on the structural units of GMX-MFe3 sample (Fig. S12(a)). Combined with the morphology of nanoparticles presented in HRTEM images (Fig. 4(e) and (f)), it can be concluded that spherical particles correspond to Fe_2C, while TiO_2 nanoparticles present elongated shapes. Moreover, for GOS-MFe (obtained by homogeneous mixing of GOS and equal amounts of ferrocene and treated by microwave irradiation), only spherical particles could be found on its structural units (Fig. S12(b)), which also proves that the elongated particles are TiO_2 generated by the in-situ oxidation of MXene. This is consistent with the conclusion obtained through the analysis of Fig. S12.

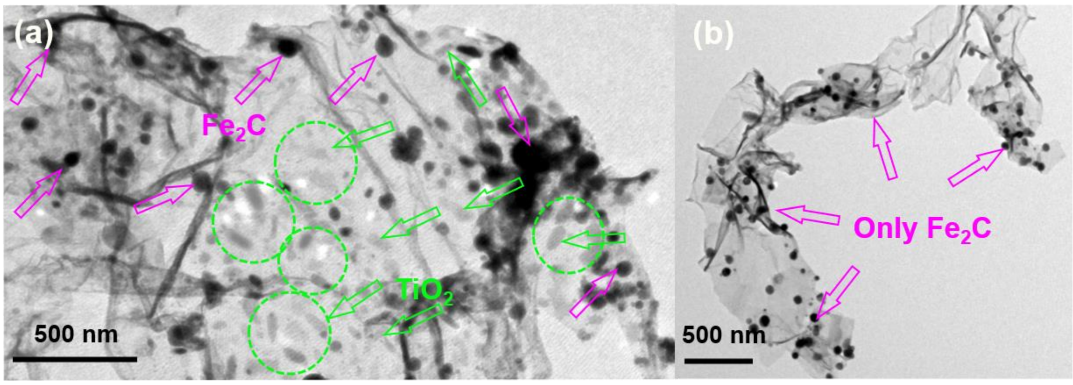

Fig. S12 TEM images of the structural units for (**a**) GMX-MFe3 and (**b**) GOS-MFe

S6 Demonstration of the Ordered Intercalation Periodicities of GO (or rGO) and MXene in Structural Units of Different Microspheres

The estimation of the periodic arrangement of the nanosheets of GO and MXene was done by assuming that the total weight of the GMM is 120 g and the GO and MXene nanosheets are similar in size [S11]. For GMX3, the mass ratio of GO and MXene is 1:1 (60:60 g). The theoretical specific surface areas of single layer GO and MXene are 736.6 m^2 g^{-1} [S12] and 483.94 m^2 g^{-1} [S13], respectively. Therefore, the total theoretical specific surface areas of GO and MXene are 44,196 and 29,036.4 m^2, respectively. The layer ratio of GO and MXene is 1.52:1, which means that, for the ideal structural unit of GMXs, the GO and MXene is stacked periodically in the form of 1-2 layers corresponding to one layer of MXene. Similarly, the layer ratios of GO: MXene are

4.57:1, 3.04:1 and 0.76:1 for GMX1, GMX2 and GMX4, respectively. The different intercalation periodicities of nanosheets would eventually be preserved in the GMX-MFe.

Furthermore, as shown in Fig. S13, the HRTEM images reflect the different intercalation periodicities of GO and MXene in the heterogeneous structural units of the GMX series microspheres, which is highly consistent with the intercalated structure of GM1-GM4 in the MGH model. Combined with the above calculations and the XRD patterns of the GMX series (Fig. 4(b)), it was confirmed that the existence of different intercalated structures corresponding to each type of microspheres is statistically significant.

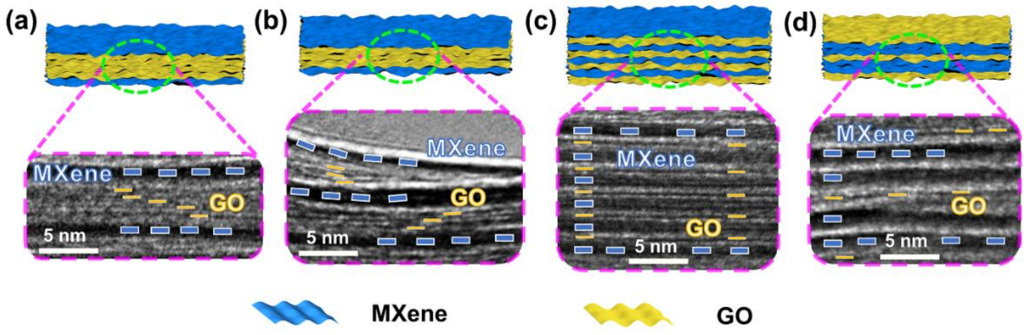

Fig. S13 Schematic illustration and corresponding HRTEM images of structural units showing the variation with GO/MXene ratio for different microspheres: (**a**) GMX1, (**b**) GMX2, (**c**) GMX3, (**d**) GMX4

S7 Interface Polarization Density Analysis

The real process of interfacial charge accumulation may be affected by many other factors like functional groups of MXene and rGO nanosheets, or the Maxwell-Wagner effect which can only explain part of the reason of interfacial charge accumulation. However, it does not prevent it from being a powerful tool for a basic understanding of charge accumulation and polarization intensity at the heterointerface. Based on MGH model, the interfacial polarization charge density should be as follows, according to Eq. (S1.8):

$$\sigma_f = \int [J_1 - J_2]\, dt = \frac{\varepsilon_2\sigma_1 - \varepsilon_1\sigma_2}{\sigma_1 d_2 + \sigma_2 d_1} \cdot U$$

$$= \frac{\varepsilon_2\sigma_1 - \varepsilon_1\sigma_2}{d_1(\sigma_1\gamma + \sigma_2)} \cdot U \qquad (S6.1)$$

where $\gamma = \frac{d_2}{d_1}$ is the defined thickness factor. The interfacial polarization charge density σ_f is not only related to the dielectric parameters (ε, σ), but also to the thickness factor γ. Based on the microstructure analysis, the thickness of MXene and rGO single nanosheet is assumed to be 1. As shown in Fig. S14, from GM1 to GM3, the thickness of MXene phase in periodic unit keeps constant $(d_1 = 1)$. As the number of rGO nanosheets decreases, the thickness factor $\gamma(\frac{d_1}{d_2})$ should be $\gamma_{GM1} > \gamma_{GM2} > \gamma_{GM3}$, so the polarization charge density should be $\sigma_{GM1} < \sigma_{GM2} < \sigma_{GM3}$. As for GM4, we can calculate that the d_1 is equal to 2 and γ_{GM4} is 1/2. By plugging d_1 and γ_{GM4} into the above equation, it can be concluded that $\sigma_{GM2} < \sigma_{GM4} < \sigma_{GM3}$. In conclusion, the interfacial polarization charge density in GM1 to GM4 should be $\sigma_{GM3} > \sigma_{GM4} >$

$\sigma_{GM2} > \sigma_{GM1}$. And the polarization interface area of different periodic intercalated units from GM1 to GM4 should be $S_{GM3} > S_{GM4} > S_{GM2} > S_{GM1}$ under the condition of fixed total nanosheets number. Combining the polarization interface area S and polarization charge density σ_f, we can conclude that the periodic intercalation model GM3 has the highest interfacial polarization intensity [S4]. However, the actual charge transfer and aggregation mechanism still need to be considered from the perspective of work function and so on, which is shown in the main manuscript.

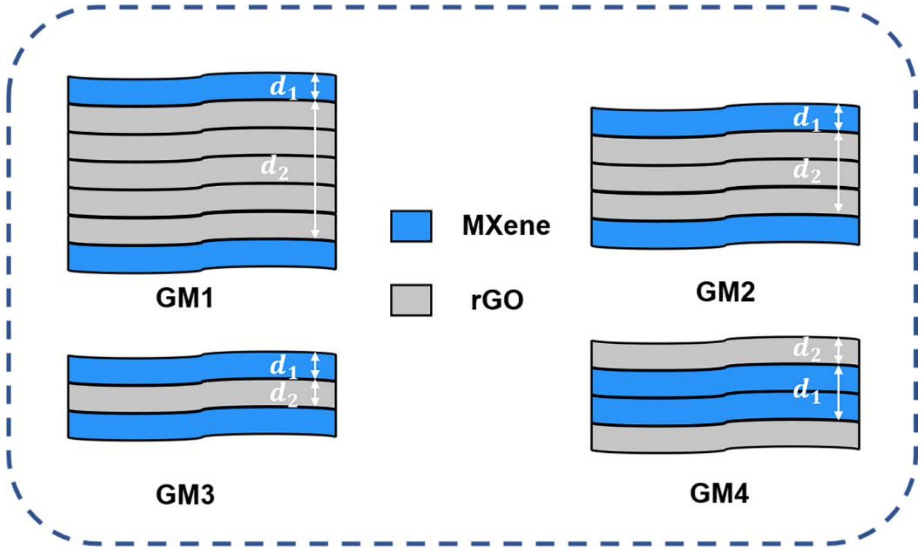

Fig. S14 Schematic diagram of MXene/rGO interface model based on component regulation

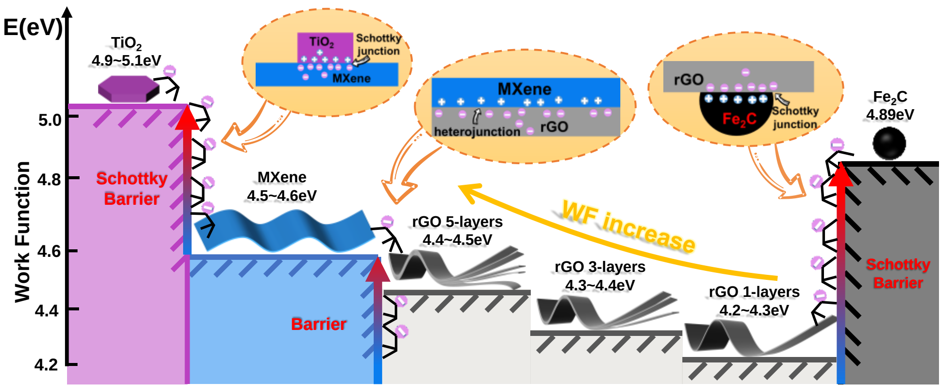

Fig. S15 The work function differences of MXene, rGO with different layers, TiO_2, Fe_2C

S8 Power Loss Distribution Simulation in CST

The power loss distribution simulation was carried out by CST microwave studio suite (2020). A template of periodic structures with metamaterial-full structure workflow was chosen for simulation. Frequency domain solver was used in this simulation process. The overall properties of this simulation are shown in Fig. S16(a). To speed up the meshing and simulation processes, we scale up the model to millimeter scale (more than 100 million-unit cells must be calculated without scale-up). The plane wave was set along the intercalation structure, which means that the incident direction of EMW was vertical to the model. The setup of solver parameters is shown in Fig. S16(c), tetrahedral mesh type was used to mesh these models.

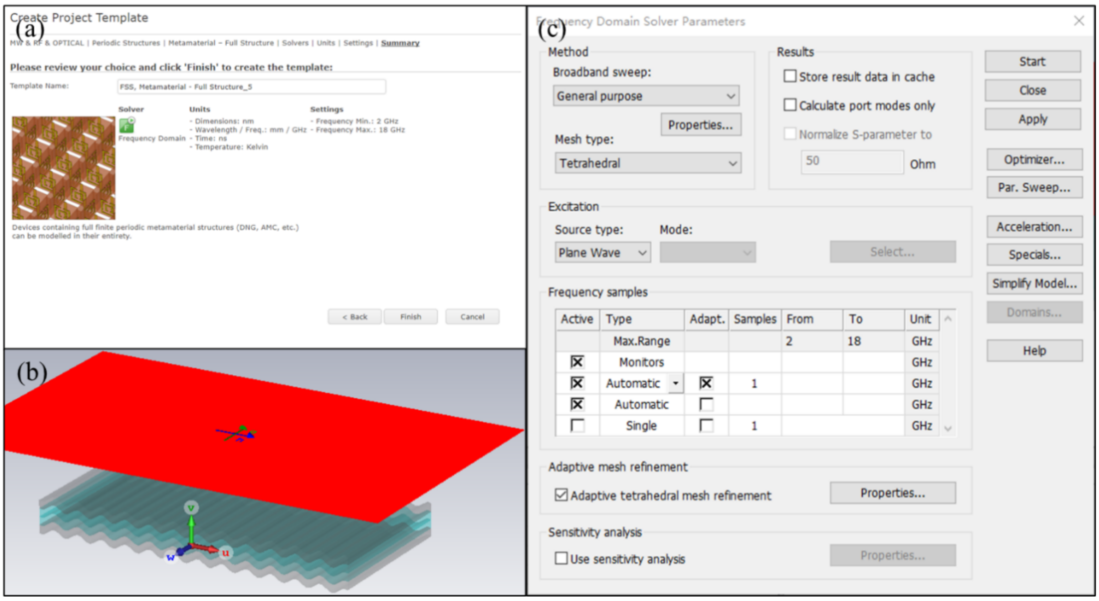

Fig. S16 (**a**) The overall parameters of the simulation project, (**b**) The model of intercalation structure in CST microwave studio, (**c**) The setup of solver parameters

S9 Permeability Analysis

The magnetic loss and dielectric loss of GMX-MFe dissipate electromagnetic wave energy synergistically. The magnetic loss typically arises from natural resonance, exchange resonance and eddy current loss. If the magnetic loss is derived from eddy current loss only, C_0 in the following equation does not vary with frequency [S5]:

$$C_0 = \mu'' \left(\mu'\right)^{-2} f^{-1} \qquad \text{(S8.1)}$$

The values of C_0 clearly fluctuate with the testing frequency for the magnetic microspheres (Fig. S17), suggesting that natural resonance (at low frequency) and exchange resonance (at high frequency) mainly contribute to magnetic loss.

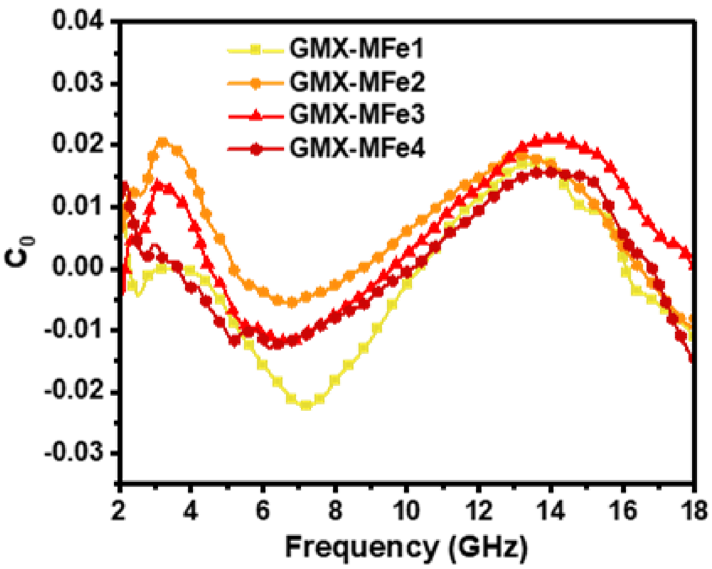

Fig. S17 C_0-f curves of GMX-MFe1, GMX-MFe2, GMX-MFe3, and GMX-MFe4

S10 Comparison of Polarization Loss Ratio with Recently Reported Works

Table S1 Comparison of polarization loss ratio with recently reported works

**Material**	ε_p'' **ratio**	**Refs.**
MXene@GO hybrid microspheres	~0.01	[S6]
RGO/Silicon Nitride Composite	~0.32	[S7]
3D flower-like Co_3O_4-MWCNT	~0.25	[S8]
Core-shell heterogeneous Carbon@RGO/Fe_3O_4	~0.03	[S9]
Honeycomb-like porous N-doped carbon confined CoP nanoparticles	~0.62	[S10]
GMX-MFe3	~0.71	This work
GMX-MFe4	~0.79	This work

S11 Electromagnetic wave absorbing performance and electromagnetic parameters

By regulating the ratio of GO and MXene in the precursor dispersion, the intercalation periodicity of the 2D components in the GMX microspheres can be regulated. Such structural difference in the stacking period is retained in the final 2D/2D/0D/0D heterojunction of GMX-MFe, which ultimately affects the EMA performance.

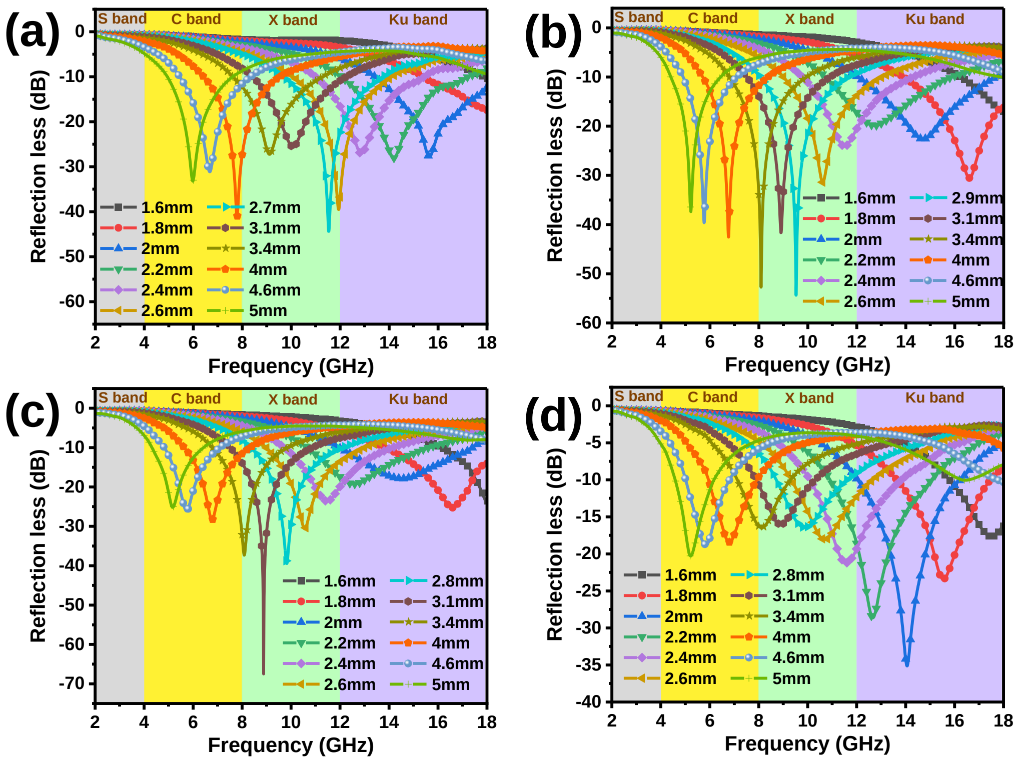

Fig. S18 2D RL curves of (**a**) GMX-MFe1, (**b**) GMX-MFe2, (**c**) GMX-MFe3 and (**d**) GMX-MFe4

The frequency corresponding to RL_{min} shifts to lower frequencies as the thickness increases, in line with the quarter-wavelength ($1/4\ \lambda$) offset theory [S11].

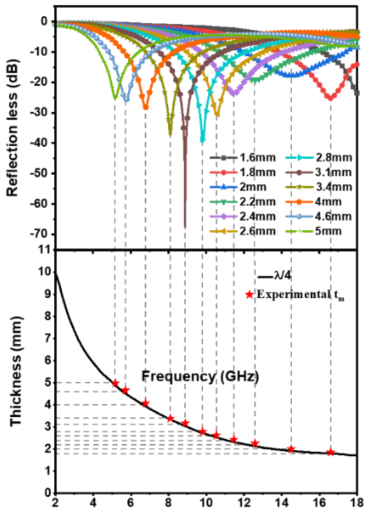

Fig. S19 The quarter wavelength thickness matching of GMX-MFe3

S12 Performance Comparison with Previously Reported Heterostructures

Table S2 shows a comparison of the obtained GMX-MFe with previously reported heterostructures. By spray-freeze-drying and microwave irradiation, the cycles of GO and MXene layers were easily adjusted by their mass ratio, resulting in excellent EMA performance at a very low filler loading of 5 wt%. In particular, the specific RL_{min} value can be optimized to -1348 dB.

Table S2 EMA performances of related 3D graphene or/and MXene based absorbers compared with this work

Absorber	Content (wt%)	RLmin (dB)	RLmin Matching Thickness (mm)	EAB (GHz)	EAB Matching thickness(mm)	Specific RL （dB）	Refs.
MXene/Fe_3O_4 Microspheres	——	-50.6	2.0	4.67	2.0	——	[S12]
RGO/MXene/Fe_3O_4 microspheres	35%	-51.2	2.9	4.7	2.9	-146.3	[S13]
MXene-rGO/CoNi films	10%	-54.1	2.1	4.98	2.1	-541	[S5]
MXene@GO hybrid microspheres	10%	-49.1	1.2	2.9	1.2	-491	[S6]
Core-shell heterogeneous Carbon@RGO/Fe_3O_4)	5%	-61	2.5	6.88	2.5	-1220	[S9]
Core–Shell Fe_3O_4@C/rGO	11%	-59.3	3.67	6.72	2.0	-539.1	[S14]
Fe_3O_4@Ti_3C_2 Tx/CNTs microspheres	20%	-40.1	2.0	5.8	2.0	200.5	[S15]
sandwich-like C/TiO_2/α-Fe	70%	-45.1	3.5	3.5	1.5	——	[S16]
Ni/TiO_2/C	60%	-39.9	1.4	3.04	1.5	-66.5	[S17]
GMX-MFe2	5%	-54.3	2.9	5.68	2.0	-1086	This work
GMX-MFe3	5%	-67.4	3.1	5.47	2.0	-1348	This work

Figure S20 shows that GMX-MFe1, GMX-MFe2 and GMX-MFe4 have values of $|Z_{in}/Z_0|$ close to 1 for the best RL_{min} values and also demonstrates that the combination of proper attenuation loss and ideal impedance matching leads to the best EMA performance among the absorbers.

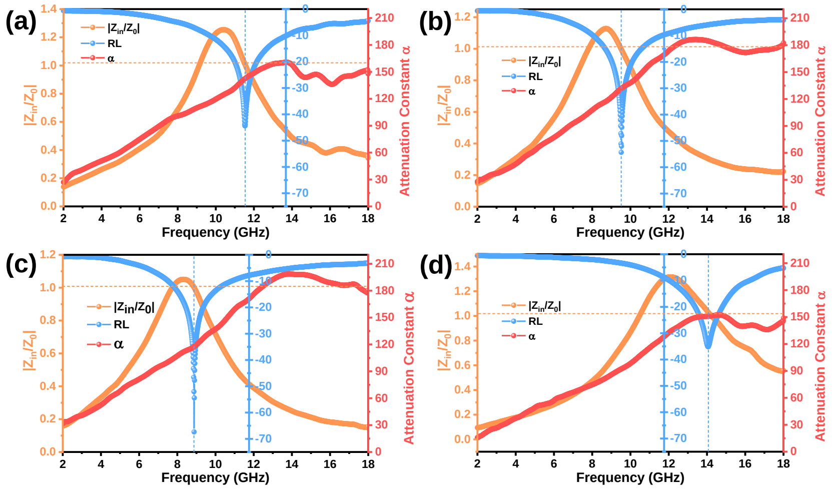

Fig. S20 Frequency-dependent RL, $|Z_{in}/Z_0|$, and α values for (**a**) GMX-MFe1, (**b**) GMX-MFe2, (**c**) GMX-MFe3, (**d**) GMX-MFe4

S13 RCS Simulation in CST

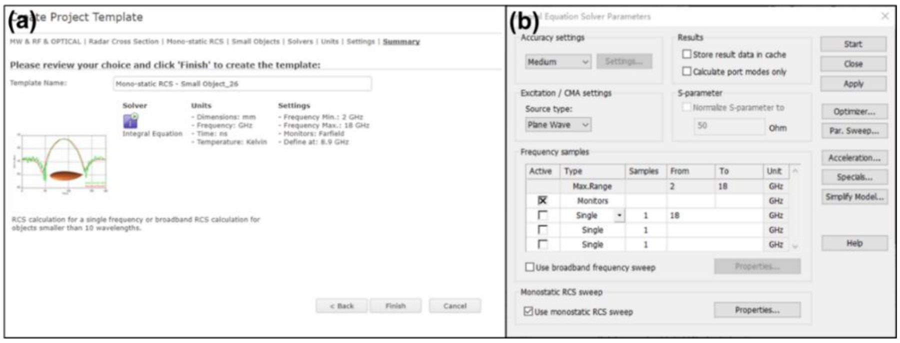

Fig. S21 (**a**) The overall parameters of the simulation project. (**b**) The setup of solver parameters

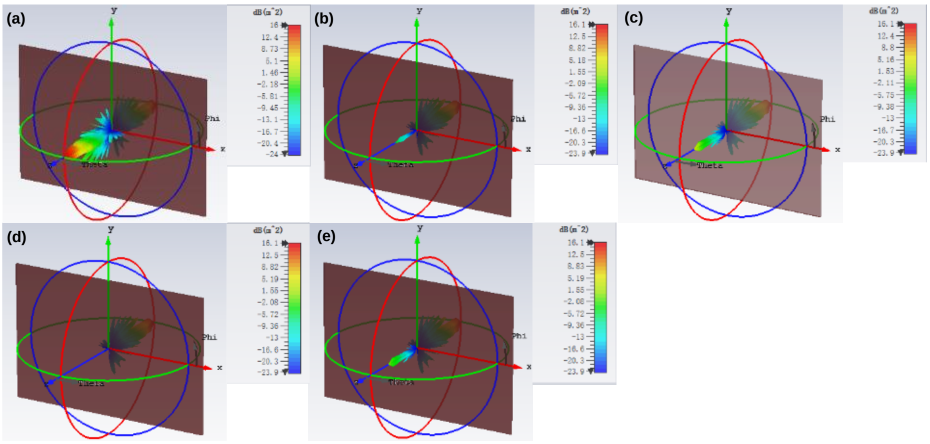

Fig. S22 3D radar wave scattering signals of (**a**) GMX3, (**b**) GMXM3, (**c**) GMX-MFe1, (**d**) GMX-MFe2, (**e**) GMX-MFe4

Figure S23 shows the 2D RCS values of GMX3 and GMX-MFe3 in polar coordinates at 8GHz, 10GHz and 12GHz. Compared with GMX3, GMX-MFe3 has significantly reduced the RCS values of PEC plate in the X band.

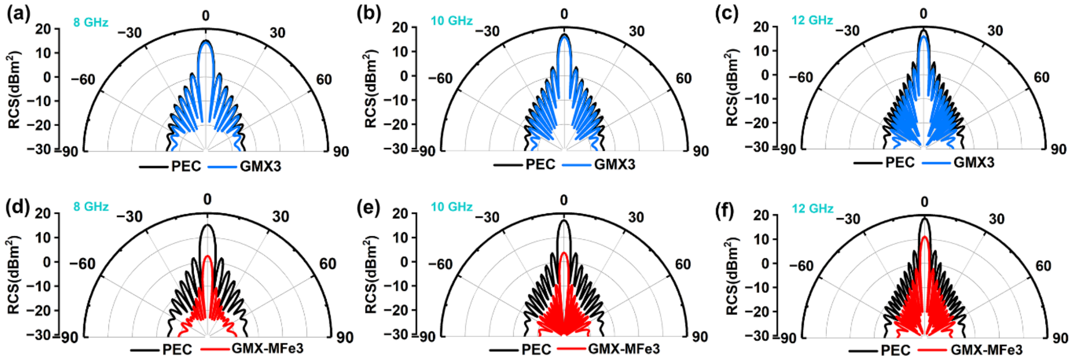

Fig. S23 RCS in polar coordinate system of GMX-3 coated PEC plate at (**a**) 8 GHz, (**b**) 10 GHz and (**c**) 12 GHz; GMX-MFe3 coated PEC plate at (**d**) 8 GHz, (**e**) 10 GHz and (**f**) 12 GHz

Supplementary Videos

Video S1 The power loss distribution of GM3 at 8.89 GHz

Supplementary References

[S1] M. Feng, Y. Feng, T. Zhang, J. Li, Q. Chen et al., Recent advances in multilayer-structure dielectrics for energy storage application. Adv. Sci. **8**(23), e2102221 (2021). https://doi.org/10.1002/advs.202102221

[S2] F. Rogti, M. Ferhat, Maxwell–Wagner polarization and interfacial charge at the multi-layers of thermoplastic polymers. J. Electrostat. **72**(1), 91-97 (2014). https://doi.org/10.1016/j.elstat.2013.11.012

[S3] Y. Karataş, T. Çetin, İ.N. Akkuş, Y. Akinay, M. Gülcan, Rh (0) nanoparticles impregnated on two-dimensional transition metal carbides,MXene, as an effective nanocatalyst for ammonia-borane hydrolysis. Int. J. Energy Res. **46**(8), 11411-11423 (2022). https://doi.org/10.1002/er.7938

[S4] Z. Wu, Z. Yang, C. Jin, Y. Zhao, R. Che, Accurately engineering 2D/2D/0D heterojunction in hierarchical $Ti_3C_2T_x$ MXene nanoarchitectures for electromagnetic wave absorption and shielding. ACS Appl. Mater. Interfaces **13**(4), 5866-5876 (2021). https://doi.org/10.1021/acsami.0c21833

[S5] X. Li, Z. Wu, W. You, L. Yang, R. Che, Self-assembly MXene-rGO/CoNi film with massive continuous heterointerfaces and enhanced magnetic coupling for superior microwave absorber. Nano-Micro Lett. **14**(1), 73 (2022). https://doi.org/10.1007/s40820-022-00811-x

[S6] Y. Li, F. Meng, Y. Mei, H. Wang, Y. Guo et al., Electrospun generation of $Ti_3C_2T_x$ MXene@graphene oxide hybrid aerogel microspheres for tunable high-performance microwave absorption. Chem. Eng. J. **391**, 123512 (2020). https://doi.org/10.1016/j.cej.2019.123512

[S7] Z. Hou, X. Yin, H. Xu, H. Wei, M. Li et al., Reduced graphene oxide/silicon nitride composite for cooperative electromagnetic absorption in wide temperature spectrum with excellent thermal stability. ACS Appl. Mater.

Interfaces **11**(5), 5364-5372 (2019). https://doi.org/10.1021/acsami.8b20023

[S8] J.-C. Shu, X.-Y. Huang, M.-S. Cao, Assembling 3D flower-like Co_3O_4-MWCNT architecture for optimizing low-frequency microwave absorption. Carbon **174**, 638-646 (2021). https://doi.org/10.1016/j.carbon.2020.11.087

[S9] D. Zhi, T. Li, Z. Qi, J. Li, Y. Tian et al., Core-shell heterogeneous graphene-based aerogel microspheres for high-performance broadband microwave absorption via resonance loss and sequential attenuation. Chem. Eng. J. **433**, 134496 (2022). https://doi.org/10.1016/j.cej.2022.134496

[S10] B. Wang, F. Huang, H. Wu, Z. Xu, S. Wang et al., Enhanced interfacial polarization of defective porous carbon confined CoP nanoparticles forming Mott—Schottky heterojunction for efficient electromagnetic wave absorption. Nano Research (2022). https://doi.org/10.1007/s12274-022-4779-3

[S11] L. Liang, Q. Li, X. Yan, Y. Feng, Y. Wang et al., Multifunctional magnetic $Ti_3C_2T_x$ MXene/graphene aerogel with superior electromagnetic wave absorption performance. ACS Nano **15**(4), 6622-6632 (2021). https://doi.org/10.1021/acsnano.0c09982

[S12] X. Li, M. Zhang, W. You, K. Pei, Q. Zeng et al., Magnetized MXene microspheres with multiscale magnetic coupling and enhanced polarized interfaces for distinct microwave absorption via a spray-drying method. ACS Appl. Mater. Interfaces **12**(15), 18138-18147 (2020). https://doi.org/10.1021/acsami.0c00935

[S13] Y. Cui, K. Yang, J. Wang, T. Shah, Q. Zhang et al., Preparation of pleated RGO/MXene/Fe_3O_4 microsphere and its absorption properties for electromagnetic wave. Carbon **172**, 1-14 (2021). https://doi.org/10.1016/j.carbon.2020.09.093

[S14] H. Zhang, Z. Jia, A. Feng, Z. Zhou, L. Chen et al., In situ deposition of pitaya-like Fe_3O_4@C magnetic microspheres on reduced graphene oxide nanosheets for electromagnetic wave absorber. Compos. B. Eng. **199**, 108261 (2020). https://doi.org/10.1016/j.compositesb.2020.108261

[S15] C. Zhang, Z. Wu, C. Xu, B. Yang, L. Wang et al., Hierarchical $Ti_3C_2T_x$ MXene/carbon nanotubes hollow microsphere with confined magnetic nanospheres for broadband microwave absorption. Small **18**(3), e2104380 (2022). https://doi.org/10.1002/smll.202104380

[S16] G. Zhao, H. Lv, Y. Zhou, X. Zheng, C. Wu et al., Self-assembled sandwich-like MXene-derived nanocomposites for enhanced electromagnetic wave absorption. ACS Appl. Mater. Interfaces **10**(49), 42925-42932 (2018). https://doi.org/10.1021/acsami.8b16727

[S17] C. Zhou, X. Wang, H. Luo, L. Deng, S. Wei et al., Rapid and direct growth of bipyramid TiO_2 from $Ti_3C_2T_x$ MXene to prepare Ni/TiO_2/C heterogeneous composites for high-performance microwave absorption. Chem. Eng. J. **383**, 123095 (2020). https://doi.org/10.1016/j.cej.2019.123095
